# Factors Associated with Prescription of Antimicrobial Drugs for Dogs and Cats, United Kingdom, 2014–2016

**DOI:** 10.3201/eid2608.191786

**Published:** 2020-08

**Authors:** David A. Singleton, Gina L. Pinchbeck, Alan D. Radford, Elena Arsevska, Susan Dawson, Philip H. Jones, Peter-John M. Noble, Nicola J. Williams, Fernando Sánchez-Vizcaíno

**Affiliations:** University of Liverpool Leahurst Campus, Neston, UK (D.A. Singleton, G.L. Pinchbeck, A.D. Radford, E. Arsevska, S. Dawson, P.H. Jones, P.-J.M. Noble, N.J. Williams);; Animal and Plant Health Agency, Shrewsbury, UK (P.H. Jones);; University of Bristol Langford Campus, Bristol, UK (F. Sánchez-Vizcaíno)

**Keywords:** Epidemiology, pets, dogs, cats, veterinary, informatics, antibacterial agents, prescriptions, antimicrobial resistance, bacteria, United Kingdom

## Abstract

Antimicrobial stewardship is a cornerstone of efforts to curtail antimicrobial resistance. To determine factors potentially influencing likelihood of prescribing antimicrobials for animals, we analyzed electronic health records for unwell dogs (n = 155,732 unique dogs, 281,543 consultations) and cats (n = 69,236 unique cats, 111,139 consultations) voluntarily contributed by 173 UK veterinary practices. Using multivariable mixed effects logistic regression, we found that factors associated with decreased odds of systemic antimicrobial prescription were client decisions focused on preventive health: vaccination (dogs, odds ratio [OR] 0.93, 95% CI, 0.90–0.95; cats, OR 0.92, 95% CI 0.89–0.95), insurance (dogs, OR 0.87, 95% CI 0.84–0.90; cats, OR 0.82, 95% CI 0.79–0.86), neutering of dogs (OR 0.90, 95% CI 0.88–0.92), and practices accredited by the Royal College of Veterinary Surgeons (OR 0.79, 95% 95% CI 0.68–0.92). This large multicenter companion animal study demonstrates the potential of preventive healthcare and client engagement to encourage responsible antimicrobial drug use.

Antimicrobial drug use is a key driver of the promotion and transmission of antimicrobial resistance in humans, livestock, and companion animals (e.g., dogs, cats) (*1*–*5*). Of these groups, the role of companion animals in the development (*1*,*2*), carriage, (*6*) and transmission of antimicrobial-resistant bacteria among animal and human populations is being increasingly realized, partly because of the close proximity in which these animals reside with humans (*5*,*7*,*8*). Indeed, companion animals are now included in recent global action plans aimed at tackling the global health threat of antimicrobial resistance (*9*).

In human medicine, electronic health records (EHRs) and qualitative research techniques have been used extensively to identify practitioner- and patient-led factors associated with the likelihood of antimicrobial drug prescription (*10*–*13*). In veterinary medicine, studies investigating antimicrobial drug prescribing practices and related risk factors are more limited (*14*). Companion animal research has largely focused on postal surveys (*15*,*16*) and in-person interviews (*17*) to explore perceptions held by veterinary practitioners. However, recent advances in veterinary health informatics have provided opportunities for widescale use of veterinary EHRs to survey antimicrobial prescription (*18*,*19*).

Thus far, key insights into antimicrobial prescription frequency and variety have been demonstrated (*20*–*23*), including an apparent increased use of cefovecin in cats (*21*,*22*); the World Health Organization considers this third-generation cephalosporin to be a highest priority critically important antimicrobial (HPCIA) (*24*). Considerable interpractice (*20*,*22*), regional (*21*), and clinical presentation (*22*,*25*,*26*) variability in antimicrobial drug prescription frequency and choice has also been identified. Although previous studies have indicated divergence of veterinary opinion over when antimicrobial therapy is justified and which classes of antimicrobial drugs would be most appropriate (*15*–*17*), the reasons why such variation exists are unknown.

To identify factors potentially influencing antimicrobial prescribing in the clinical environment, we analyzed EHRs for a large, diverse population of dogs and cats, collected from a network of volunteer first-opinion (general) veterinary practices across Great Britain (England, Scotland, and Wales). We explored associations between antimicrobial prescription (including antimicrobials authorized for systemic administration, antimicrobials authorized for topical administration, and HPCIAs) and a range of veterinary practice, practitioner, client, and animal-related factors (including socioeconomic factors and preventive healthcare interventions) for animals presented primarily for investigation of disease.

## Materials and Methods

### Data Collection

For this cross-sectional study, we examined EHRs from 178 volunteer veterinary practices (386 unique sites) taking part in the Small Animal Veterinary Surveillance Network (SAVSNET; University of Liverpool, Liverpool, UK; ethical approval reference no. RETH000964), by using the Robovet practice management system (Covetrus, https://www.covetrus.com). We retrieved EHRs from booked consultations (*19*) from April 1, 2014, through March 31, 2016. Each consultation record included patient species, breed, sex, neuter status, insurance status, microchip status, vaccination history, date of birth, client’s postal code, and any products dispensed at time of consultation. Every consultation record was further classified by the attending veterinary professional into 1 of 10 main presenting complaints (categorized as healthy, unhealthy, or postoperative), indicating the main reason the animal was presented to the veterinary practice, as previously described (*22*).

### Data Management

#### General Data Management

Initially available were consultations for 762,648 dogs and 300,606 cats. We excluded animals for whom dates of birth were probably incorrectly recorded (i.e., 1,577 dogs recorded as >24.5 years and 2,467 cats as >26.0 years of age at consultation) and animals for whom a valid client postal code was lacking (23,705 dogs, 9,901 cats). We included only consultations in which animals were recorded as unhealthy (sick animal consultations) according to main presenting complaint (MPC) (282,263 of 737,366 remaining dog and 111,367 of 288,238 remaining cat consultations). We also excluded 5 veterinary practices that provided insufficient EHRs (<50 consultations) for adequate statistical analyses. 

Using a semiautomated rule-based text-mining method as previously described (*22*), we identified antimicrobial prescription via the text-based product description and classified use as systemic (oral or injectable) or topical (topical, aural, ocular). All fluoroquinolones, macrolides, and third-generation cephalosporins were considered HPCIAs (*24*). We compiled a list of antimicrobials authorized for use in dogs or cats use in the United Kingdom (Appendix Table 1).

#### Animal Factors

Animals were considered vaccinated if the most recently recorded vaccination date (disregarding vaccine composition) was <3.5 years (broadly reflective of current vaccine interval guidelines) before the relevant consultation date (*27*). Breeds were summarized according to standardized breed terms (*28*) before being categorized into either genotypically similar breed groups (*29*), crossbreeds, breeds not yet genetically classified (unclassified), or breed not recorded/recognizable (unknown).

#### Client Factors

Using clients’ home postal code, we assigned a measure of predicted deprivation to each client according to the most recent Indices of Multiple Deprivation (IMD): England 2015 (https://www.gov.uk/government/statistics/english-indices-of-deprivation-2015), Scotland 2012 (https://www2.gov.scot/Topics/Statistics/SIMD/DataAnalysis/Background-Data-2012), and Wales 2014 (https://statswales.gov.wales/Catalogue/Community-Safety-and-Social-Inclusion/Welsh-Index-of-Multiple-Deprivation/Archive/WIMD-2014). Because IMD measures between countries are not directly comparable, country was included in statistical models as a 3-level factor, and each country’s complete set of IMD ranks was rescaled to the range of 0 to 1, with 1 corresponding to the least deprived area.

We determined country of residence and urban/rural status by referring to the National Statistics Postcode Look-up (https://geoportal.statistics.gov.uk/datasets/4f71f3e9806d4ff895996f832eb7aacf). The recorded centroid associated with each postal code was used to place each client within a 1-km^2^ gridded cell, and each EHR was hence associated with an estimate of the number of dogs or cats within each 1-km^2^ gridded cell as defined by Aegerter et al. (*30*). We then used postal code district to provide an estimate of the number of dogs or cats per household for each recorded postal code (*30*).

#### Veterinary Practice and Practitioner Factors

We used the Royal College of Veterinary Surgeons (RCVS) Practice Register (https://findavet.rcvs.org.uk/home, accessed 2016 Oct 18) to summarize each veterinary practice into 4 categories of advertised species treated: companion animal; mixed (companion animal, large animal, and equine); companion and large animal; and companion animal and equine. Practices were considered accredited under the voluntary RCVS Practice Standards Scheme if >1 site was recorded as accredited (Core Standards, General Practice, or Veterinary Hospital), and as an RCVS Veterinary Hospital if the practice contained a veterinary hospital site. We also recorded practices listing referrals as an interest and practices employing >1 veterinary surgeon holding RCVS Advanced Veterinary Practitioner status or separate RCVS Specialist status in areas of relevance to companion animals.

### Statistical Analyses

We used R (https://www.r-project.org) for all analyses. Descriptive proportions and 95% CIs were adjusted for clustering within sites (bootstrap method, n = 5,000 samples). Using the R package lme4 (https://cran.r-project.org/web/packages/lme4/index.html), we fitted univariable and multivariable mixed effects logistic regression models separately for dogs and cats. Because likelihood ratio tests (LRTs), the Akaike Information Criterion (AIC), the Bayesian Information Criterion (BIC), and evidence of interpractice antimicrobial prescription frequency variation (*22*) indicated that observations were clustered within veterinary practice, site, and animal, we therefore included all 3 factors as random intercepts in all models. We conducted separate analyses to assess the association between explanatory variables and 3 binary outcomes of interest: antimicrobial prescription authorized for systemic administration (systemic antimicrobial), topical administration (topical antimicrobial), and systemically administered HPCIAs.

Initial univariable screening included 15 categorical variables (sex, neutered status, microchip status, insurance status, vaccination status, genetic breed group, country of residence, client urban/rural status, main presenting complaint, treated species [practice type], RCVS accreditation, RCVS Veterinary Hospital, referral interest, RCVS Advanced Veterinary Practice, and RCVS specialist), and 4 continuous variables (age at consultation, rescaled IMD [rIMD] rank, dog or cat population per square kilometer, and mean number of dogs or cats per household at district of residence). For continuous explanatory variables, we included up to cubic polynomial terms if an LRT, AIC, and BIC indicated significantly improved fit, compared with linear and lesser polynomial terms. Explanatory variables were retained for multivariable analysis if an LRT indicated p<0.20 against a null model.

To minimize AIC and BIC, we conducted manual stepwise backward elimination on multivariable models. A 2-way interaction between rIMD and the 3-level factor country was included in the initial multivariable model (and were deleted if AIC and BIC decreased); country alone was a false intercept. Confounding was accounted for by assessing effect variation upon removal of variables. Two-way interaction terms between other explanatory variables were assessed by using AIC, BIC, and an LRT. The variance inflation factor was used to assess multicollinearity (https://CRAN.R-project.org/package=car). For continuous variables, projected prescription probabilities and associated 95% CIs were calculated from log odds by using sjPlot (https://cran.r-project.org/web/packages/sjPlot/index.html). Statistical significance was defined as p<0.05.

## Results

Analyzed data were from 281,543 consultations for sick dogs (155,732 unique dogs) and 111,139 sick cats (69,236 unique cats) from 173 veterinary practices (379 sites). A descriptive population summary is shown in Table 1, and a summary of genetic breed groups included in this study is shown in Appendix Table 2.

**Table 1:** Descriptive demographic summary of consultations for sick dogs and cats in study of factors associated with prescription of antimicrobial drugs for dogs and cats, United Kingdom, 2014–2016*

Category	Dogs, n = 281,543	Cats, n = 111,139	
Categorical factors			
Country			
England	86.6 (81.4–91.9)	88.6 (83.8–93.5)	
Scotland	6.1 (3.0–9.1)	4.5 (2.1–6.9)	
Wales	7.4 (2.8–12.0)	7.0 (2.1–6.9)	
Sex			
M	51.8 (51.3–52.3)	51.8 (51.3–52.4)	
F	48.2 (47.7–48.7)	48.2 (47.6–48.7)	
Neutered	64.6 (63.3–65.9)	82.8 (81.7–84.0)	
Microchipped	54.4 (52.4–56.3)	37.8 (36.0–39.5)	
Vaccinated	70.0 (68.6–71.3)	52.7 (51.2–54.1)	
Insured	33.5 (31.1–35.9)	19.3 (17.3–21.3)	
Urban	63.8 (59.5–68.1)	70.2 (66.2–74.2)	
Main presenting complaint			
Gastroenteric	11.3 (11.0–11.6)	8.3 (8.0–8.7)	
Respiratory	4.0 (3.8–4.1)	5.5 (5.2–5.8)	
Pruritus	18.0 (17.3–18.6)	10.3 (9.9–10.7)	
Trauma	16.8 (16.1–17.5)	17.0 (16.3–17.7)	
Tumor	6.0 (5.8–6.3)	3.9 (3.6–4.1)	
Kidney disease	0.7 (0.6–0.8)	2.9 (2.5–3.2)	
Other unwell	43.3 (42.0–44.6)	52.1 (50.9–53.4)	
Practice type			
Mixed	22.7 (15.1–30.3)	18.1 (11.6–24.6)	
Companion animal	70.6 (62.4–78.8)	76.0 (68.9–83.1)	
Companion and equine	2.4 (0.7–4.0)	2.3 (0.7–4.0)	
Companion and large	4.3 (0.4–8.2)	3.5 (0.3–6.8)	
Accredited	83.9 (77.1–90.6)	83.5 (76.5–90.5)	
Hospital status	20.2 (14.4–26.0)	20.0 (14.5–25.5)	
Referral interest	27.9 (20.9–34.9)	27.3 (20.3–34.2)	
Employed RCVS AVP†	24.5 (17.2–31.7)	26.7 (19.2–34.2)	
Employed RCVS specialist†	2.5 (0.8–4.2)	1.9 (0.6–3.1)	
Continuous factors			
Age at consultation			
Mean	7.1 (7.1–7.2)	9.5 (9.5–9.6)	
Median (min–max)	7.2 (0–22)	9.7 (0–25.9)	
Rescaled indices of multiple, mean	0.59 (0.59–0.60)	0.60 (0.60–0.61)
rIMD deprivation rank, median (min–max)	0.62 (0.0–1.0)	0.63 (0.0–1.0)	
Animals/household (*30*)			
Mean	0.59 (0.59–0.59)	0.50 (0.49–0.50)	
Median (min–max)	0.47 (0–6.0)	0.39 (0–3.6)	
Animals/km^2^ (*30*)			
Mean	399.4 (397.8–401.0)	409.4 (407.0–411.8)	
Median (min–max)	266 (0–4,360)	288 (0–5,363)	

*Values are % consults (95% CI) except as indicated. AVP, Advanced Veterinary Practitioner; max, maximum; min, minimum; rIMD, rescaled Indices of Multiple Deprivation; RCVS, Royal College of Veterinary Surgeons; †At least 1 employed veterinary surgeon holding RCVS AVP status, specialist status, or both.

### Dogs

#### Prescription of Antimicrobial Drugs

Systemic antimicrobial drugs were prescribed during 25.7% (95% CI 24.9%–26.6%) of consultations, topical antimicrobials during 14.2% (95% CI 13.9%–14.6%), and systemic HPCIAs during 1.4% (95% CI 1.2%–1.6%). The most commonly prescribed class of systemic HPCIAs was fluoroquinolones (0.9% of sick animal consultations, 95% CI 0.7%–1.0%), followed by third-generation cephalosporins (0.5%, 95% CI 0.4%–0.6%) and macrolides (0.1%, 95% CI 0.0%–0.2%). Antimicrobial prescription summarized according to common consultation by breed is shown in Appendix Table 3.

#### Prescription of Systemic Antimicrobial Drugs

**Figure 1:**
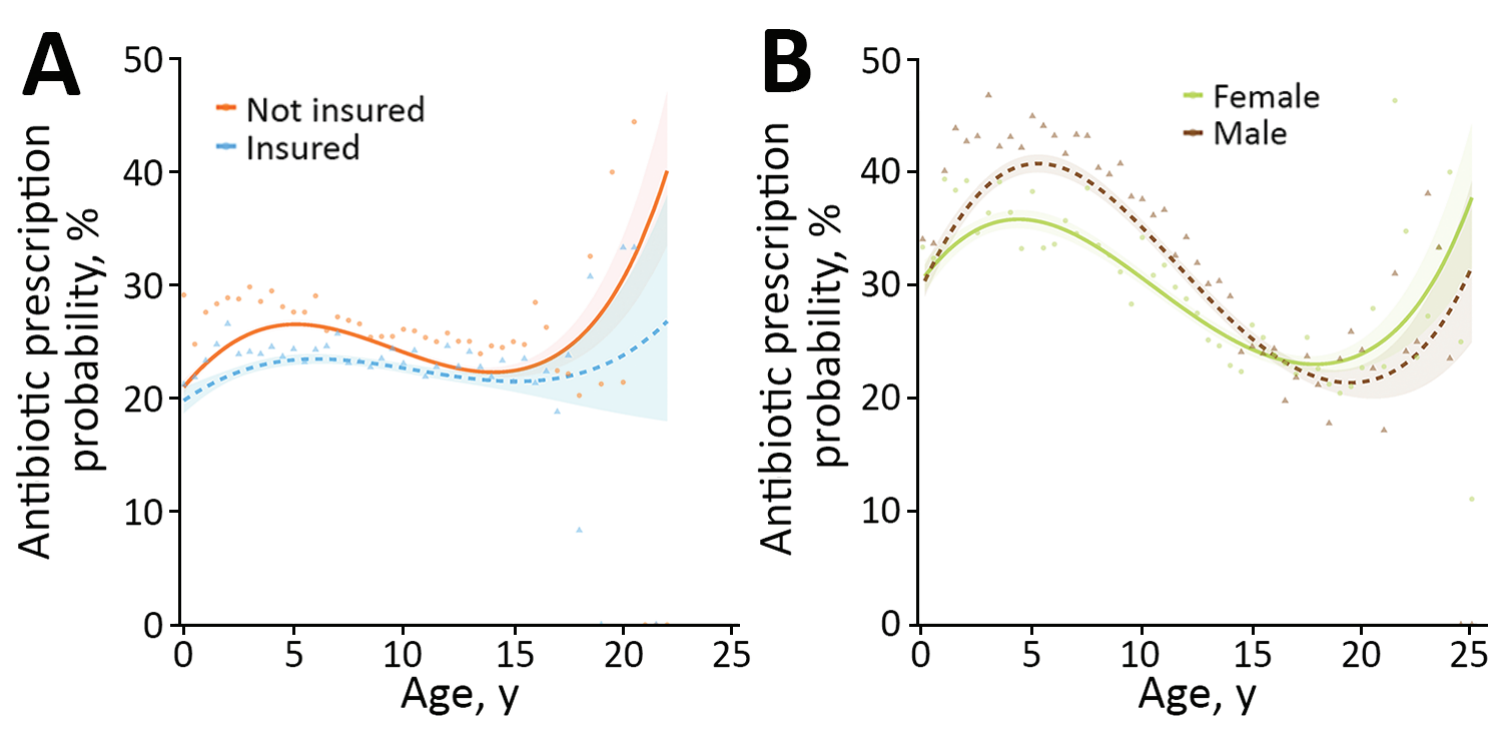
Results from 2 multivariable mixed effect logistic regression models predicting probability of systemic antimicrobial prescription in study of factors associated with prescription of antimicrobial drugs for dogs and cats, United Kingdom, 2014–2016. Modeling is shown for sick dogs (A) and cats (B) against age of the animal at time of consultation, in years. For dogs, an interaction term considering current insurance status has been included; for cats, an interaction term considering sex has been included. Lines refer to predicted probability; shading relates to 95% CIs for such predictions. Points and triangles are plotted to show original data points expressing the percentage of animals of each relevant age group (rounded to 0.5-year groups) for which a systemic antimicrobial was prescribed, according to the dataset analyzed.

Descriptive analyses and univariable model results are summarized in Appendix Table 4. Final multivariable model results are available in Table 2. Systemic antimicrobial drugs were less likely to be prescribed for vaccinated or neutered dogs than for nonvaccinated or non-neutered dogs. Systemic antimicrobial drugs were also less likely to be prescribed for insured dogs up to ≈12 years of age (Figure 1, panel A). Odds for prescription of a systemic antimicrobial drug were greater for dogs with an MPC that was respiratory than for those with a gastroenteric MPC. Mixed practices were associated with significantly increased odds of this prescription compared with practices treating companion animals only. RCVS-accredited practices were less likely to prescribe a systemic antimicrobial.

**Table 2:** Results from a multivariable mixed effect logistic regression model assessing the association between a range of categorical animal, owner, practitioner and practice-related factors and the probability of prescribing a systemic antimicrobial for dogs, United Kingdom, 2014–2016*

Category	β	SE	OR (95% CI)	p value
Intercept				
England	−0.08	0.08	0.93 (0.80–1.08)	NA
Scotland	−0.06	0.09	0.94 (0.79–1.12)	NA
Wales	−0.13	0.09	0.88 (0.73–1.05)	NA
Categorical factors				
Initial complaint				
Gastroenteric	NA	NA	Referent	NA
Kidney disease	−0.38	0.06	0.68 (0.61–0.76)	**<0.01**
Other unwell	−0.94	0.02	0.39 (0.38–0.40)	**<0.01**
Pruritus	−0.68	0.02	0.51 (0.49–0.53)	**<0.01**
Respiratory	0.10	0.03	1.11 (1.06–1.17)	**<0.01**
Trauma	−0.89	0.02	0.41 (0.40–0.43)	**<0.01**
Tumor	−1.18	0.03	0.31 (0.29–0.32)	**<0.01**
Neuter status				
Not neutered	NA	NA	Referent	NA
Neutered	−0.11	0.01	0.90 (0.88–0.92)	**<0.01**
Sex				
F	NA	NA	Referent	NA
M	−0.03	0.01	0.97 (0.95–0.99)	**0.01**
Vaccination status				
Not vaccinated	NA	NA	Referent	NA
Vaccinated	−0.08	0.01	0.93 (0.90–0.95)	**<0.01**
Insurance status				
Not insured	NA	NA	Referent	NA
Insured	−0.14	0.02	0.87 (0.84–0.90)	**<0.01**
Genetic breed group (*29*)				
Retriever	NA	NA	Referent	NA
Ancient/spitz	0.25	0.05	1.28 (1.17–1.40)	**<0.01**
Crossbreed	0.06	0.02	1.06 (1.03–1.10)	**<0.01**
Herding	0.14	0.03	1.15 (1.09–1.22)	**<0.01**
Mastiff-like	0.15	0.02	1.16 (1.11–1.21)	**<0.01**
Scent hound	0.10	0.04	1.11 (1.03–1.19)	**<0.01**
Sight hound	0.31	0.04	1.36 (1.25–1.48)	**<0.01**
Small terrier	0.16	0.02	1.18 (1.13–1.22)	**<0.01**
Spaniel	0.16	0.02	1.17 (1.13–1.22)	**<0.01**
Toy	−0.00	0.03	1.00 (0.94–1.05)	0.92
Unclassified	0.11	0.02	1.12 (1.07–1.16)	**<0.01**
Unknown	0.09	0.05	1.09 (0.99–1.21)	0.075
Working dog	0.19	0.03	1.21 (1.15–1.27)	**<0.01**
Practice type				
Companion animal	NA	NA	Referent	NA
Mixed	0.14	0.07	1.15 (1.01–1.30)	**0.04**
Companion and equine	−0.05	0.15	0.95 (0.71–1.27)	0.73
Companion and large	0.13	0.14	1.14 (0.86–1.50)	0.37
Accreditation status				
None	NA	NA	Referent	NA
>1 accredited site	−0.24	0.08	0.79 (0.68–0.92)	**<0.01**
Referral interest				
No	NA	NA	Referent	NA
Yes	−0.10	0.05	0.91 (0.82–1.00)	0.06
Continuous factors				
Age				
Linear	−1.12	0.01	0.89 (0.87–0.91)	**<0.01**
Quadratic	−0.09	0.01	0.92 (0.90–0.93)	**<0.01**
Cubic	0.05	0.01	1.05 (1.04–1.07)	**<0.01**
Interaction terms				
Insurance status (insured) and age				
Linear age interaction	0.08	0.02	1.09 (1.04–1.14)	**<0.01**
Quadratic age interaction	0.03	0.01	1.03 (1.00–1.06)	**0.03**
Cubic age interaction	−0.03	0.01	0.97 (0.95–1.00)	**0.02**

*n = 72,436/281,543 sick consultations. Random effect variance (± SD): animal 0.57 (± 0.76), site 0.05 (0.23), practice 0.06 (0.24). Boldface indicates significance (p<0.05). NA, not applicable; OR, odds ratio.

#### Prescription of Systemic HPCIAs

**Figure 2:**
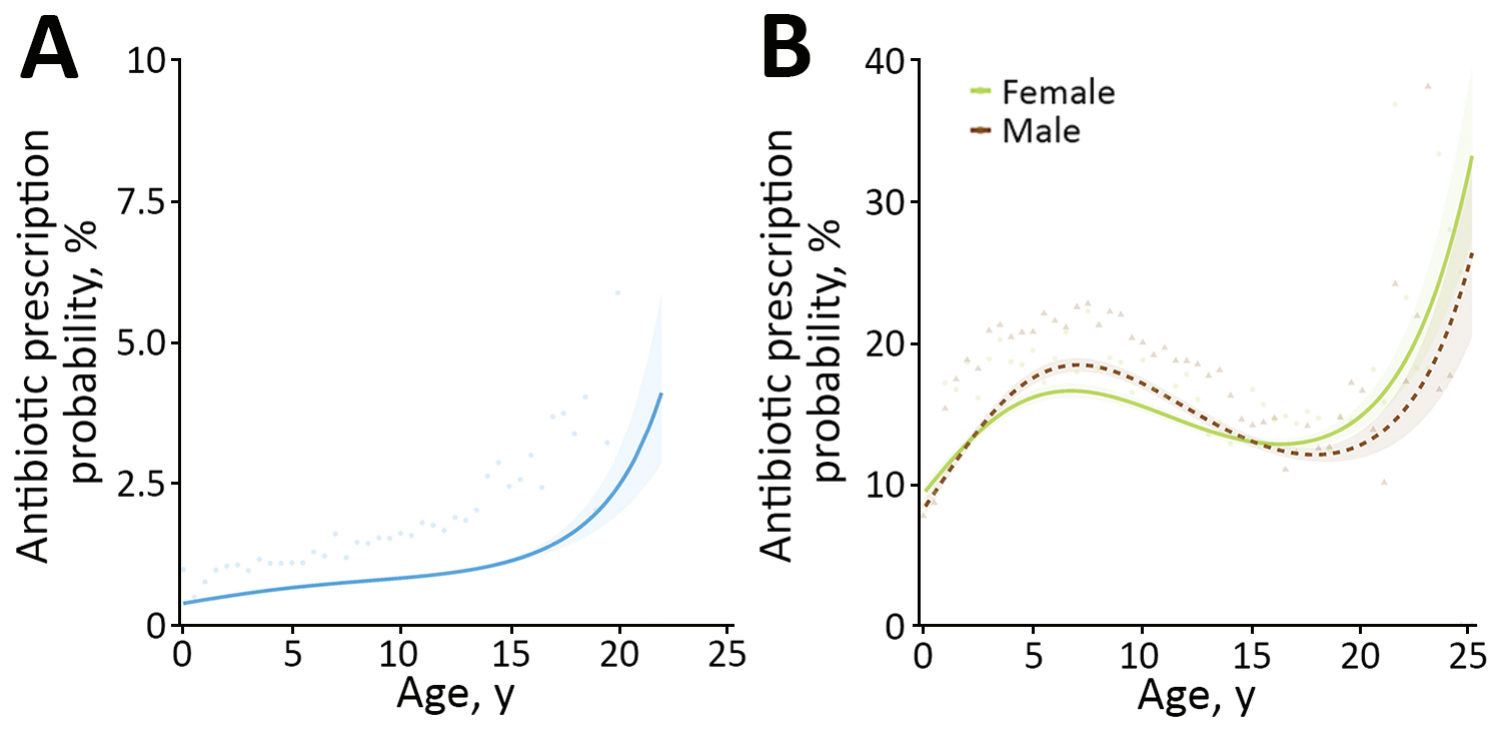
Results from 2 multivariable mixed effect logistic regression models predicting probability of systemic highest priority critically important antimicrobial (HPCIA) prescription in study of factors associated with prescription of antimicrobial drugs for dogs and cats, United Kingdom, 2014–2016. Modeling is shown for sick dogs (A) and cats (B) against age of the animal at time of consultation, in years. For cats, an interaction term considering sex has been included. Lines refer to predicted probability; shading relates to 95% CIs for such predictions. Points and triangles are plotted to show original data points expressing the percentage of animals of each relevant age group (rounded to 0.5-year groups) for which a systemic HPCIA was prescribed, according to the dataset analyzed.

Descriptive analyses and univariable model results are summarized in Appendix Table 5. Final multivariable model results are available in Table 3. Systemic HPCIAs were less likely to be prescribed for vaccinated or insured dogs; highest odds for prescription were for dogs with a respiratory MPC. Odds increased with age (Figure 2, panel A). In terms of genetic breed, the greatest odds of systemic HPCIA prescription were for the toy breed group, compared with retrievers.

**Table 3:** Results from a multivariable mixed effect logistic regression model assessing the association between a range of categorical animal, owner, practitioner and practice-related factors and the probability of prescribing a systemic highest priority critically important antimicrobial drug for dogs, United Kingdom, 2014–2016*

Category	β	SE	OR (95% CI)	p value
Intercept				
England	−4.77	0.11	0.01 (0.01–0.01)	NA
Scotland	−4.91	0.21	0.01 (0.01–0.01)	NA
Wales	−4.88	0.22	0.01 (0.01–001)	NA
Categorical factors				
Main presenting complaint				
Gastroenteric	NA	NA	Referent	NA
Kidney disease	0.11	0.18	1.12 (0.78–1.60)	0.55
Other unwell	−0.33	0.06	0.72 (0.64–0.80)	**<0.01**
Pruritus	−0.23	0.07	0.79 (0.70–0.90)	**<0.01**
Respiratory	0.29	0.09	1.33 (1.13–1.57)	**<0.01**
Trauma	−1.16	0.08	0.31 (0.27–0.37)	**<0.01**
Tumor	−0.92	0.11	0.40 (0.32–0.49)	**<0.01**
Vaccination status				
Not vaccinated	NA	NA	Referent	NA
Vaccinated	−0.10	0.04	0.91 (0.83–0.99)	**0.03**
Insurance status				
Not insured	NA	NA	Referent	NA
Insured	0.15	0.05	1.16 (1.07–1.27)	**<0.01**
Genetic breed group (*29*)				
Retriever	NA	NA	Referent	NA
Ancient/spitz	0.12	0.22	1.13 (0.73–1.74)	0.60
Crossbreed	0.24	0.08	1.27 (1.09–1.48)	**<0.01**
Herding	0.04	0.12	1.04 (0.82–1.32)	0.73
Mastiff-like	0.16	0.10	1.17 (0.97–1.43)	0.11
Scent hound	0.67	0.13	1.96 (1.52–2.52)	**<0.01**
Sight hound	0.43	0.17	1.54 (1.10–2.15)	**0.01**
Small terrier	0.67	0.08	1.96 (1.67–2.29)	**<0.01**
Spaniel	0.45	0.08	1.57 (1.33–1.84)	**<0.01**
Toy	0.94	0.10	2.56 (2.10–3.12)	**<0.01**
Unclassified	0.39	0.09	1.47 (1.24–1.74)	**<0.01**
Unknown	0.23	0.22	1.25 (0.81–1.94)	0.31
Working dog	0.45	0.11	1.56 (1.27–1.93)	**<0.01**
Continuous factors				
Age				
Linear	0.19	0.04	1.21 (1.12–1.31)	**<0.01**
Quadratic	−0.06	0.03	0.95 (0.90–0.99)	**0.03**
Cubic	0.04	0.02	1.04 (1.01–1.08)	**0.01**

*n = 3,971/281,543 sick consultations. Random effect variance (± SD): animal 3.04 (1.74), site 0.13 (0.36), practice 0.44 (0.66). Significant (p<0.05) results are displayed in boldface. NA, not applicable; OR, odds ratio.

#### Prescription of Topical Antimicrobial Drugs

**Figure 3:**
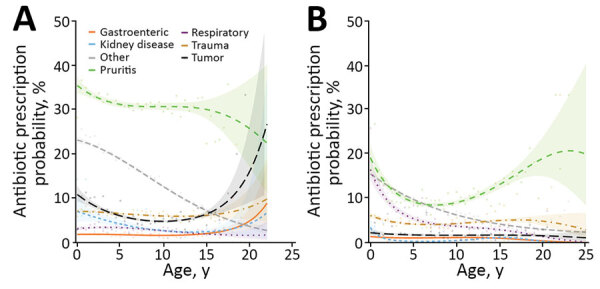
Results from 2 multivariable mixed effect logistic regression models predicting probability of topical antimicrobial prescription in study of factors associated with prescription of antimicrobial drugs for dogs and cats, United Kingdom, 2014–2016. Modeling is shown for sick dogs (A) and cats (B) against age of the animal at time of consultation, in years. For both species, an interaction term considering main reason for visit (main presenting complaint) has been included. Lines refer to predicted probability; shading relates to 95% CIs for such predictions. Points are plotted to show original data points expressing the percentage of animals of each relevant age group (rounded to 0.5-year groups) for which a topical antimicrobial was prescribed, according to the dataset analyzed.

Descriptive analyses and univariable model results are summarized in Appendix Table 6. Final multivariable model results are available in Table 4. Topical antimicrobial drugs were less likely to be prescribed for insured dogs, although odds increased significantly for male, microchipped, or vaccinated dogs. The effect of age varied according to MPC; an MPC of pruritus was generally associated with greatest odds of topical antimicrobial drug prescription throughout life, broadly decreasing with increased age (Figure 3, panel A). Odds of topical antimicrobial drug prescription were lowest for sight hounds compared with retrievers. Practices employing RCVS specialists were less likely to prescribe a topical antimicrobial.

**Table 4:** Results from a multivariable mixed effect logistic regression model assessing the association between a range of categorical animal, owner, practitioner and practice-related factors and the probability of prescribing a topical antimicrobial in dogs, United Kingdom, 2014–2016*

Category	β	SE	OR (95% CI)	p value
Intercept				
England	−4.01	0.07	0.02 (0.02–0.02)	NA
Scotland	−3.88	0.09	0.02 (0.02–0.02)	NA
Wales	−4.06	0.09	0.02 (0.01–0.02)	NA
Categorical factors				
Main presenting complaint				
Gastroenteric	-	NA	1.00	NA
Kidney disease	0.71	0.22	2.03 (1.31–3.15)	**<0.01**
Other unwell	2.41	0.07	11.18 (9.78–12.79)	**<0.01**
Pruritus	3.24	0.07	25.64 (22.39–29.35)	**<0.01**
Respiratory	0.63	0.11	1.88 (1.50–2.34)	**<0.01**
Trauma	1.35	0.07	3.87 (3.36–4.46)	**<0.01**
Tumor	1.15	0.08	3.16 (2.68–3.73)	**<0.01**
Sex				
F	NA	NA	1.00	NA
M	0.07	0.01	1.08 (1.05–1.10)	**<0.01**
Microchip status				
Not microchipped	NA	NA	1.00	NA
Microchipped	0.03	0.01	1.03 (1.00–1.06)	**0.02**
Vaccination status				
Not vaccinated	NA	NA	1.00	NA
Vaccinated	0.08	0.02	1.08 (1.05–1.11)	**<0.01**
Insurance status				
Not insured	NA	NA	1.00	NA
Insured	−0.10	0.02	0.90 (0.88–0.93)	**<0.01**
Genetic breed group (*29*)				
Retriever	NA	NA	1.00	NA
Ancient/spitz	−0.14	0.06	0.87 (0.77–0.97)	**0.02**
Crossbreed	−0.21	0.02	0.81 (0.78–0.84)	**<0.01**
Herding	−0.57	0.04	0.57 (0.53–0.61)	**<0.01**
Mastiff-like	−0.03	0.03	0.97 (0.93–1.03)	0.32
Scent hound	−0.25	0.04	0.78 (0.71–0.85)	**<0.01**
Sight hound	−0.92	0.07	0.40 (0.34–0.46)	**<0.01**
Small terrier	−0.29	0.03	0.75 (0.71–0.79)	**<0.01**
Spaniel	0.04	0.02	1.04 (1.00–1.09)	0.08
Toy	−0.14	0.03	0.87 (0.82–0.93)	**<0.01**
Unclassified	−0.06	0.03	0.94 (0.89–0.99)	**0.011**
Unknown	−0.31	0.06	0.74 (0.65–0.83)	**<0.01**
Working dog	−0.21	0.03	0.81 (0.76–0.87)	**<0.01**
Hospital status				
None	NA	NA	1.00	NA
>1 hospital site	0.06	0.04	1.07 (0.98–1.16)	0.15
Employed RCVS AVP				
None	NA	NA	1.00	NA
>1 AVP	0.08	0.04	1.08 (0.99–1.17)	0.08
Employed RCVS specialists				
None	NA	NA	1.00	NA
+ specialist	−0.27	0.09	0.77 (0.64–0.92)	**<0.01**
Continuous factors				
Age				
Linear	−0.10	0.09	0.91 (0.76–1.09)	0.30
Quadratic	0.04	0.04	1.04 (0.98–1.13)	0.39
Cubic	0.04	0.04	1.04 (0.96–1.13)	0.30
Interaction terms				
Main presenting complaint and age				
Linear age interaction				
Kidney disease	−0.33	0.27	0.72 (0.42–1.22)	0.22
Other unwell	−0.30	0.10	0.74 (0.61–0.89)	**<0.01**
Pruritus	0.08	0.10	1.08 (0.89–1.31)	0.42
Respiratory	−0.01	0.15	0.90 (0.66–1.21)	0.47
Trauma	0.01	0.10	1.01 (0.82–1.23)	0.95
Tumor	−0.15	0.12	0.86 (0.69–1.08)	0.20
Quadratic age interaction				
Kidney disease	0.04	0.15	1.04 (0.77–1.40)	0.79
Other unwell	−0.11	0.05	0.90 (0.82–0.98)	**0.02**
Pruritus	−0.00	0.05	1.00 (0.91–1.09)	0.96
Respiratory	−0.12	0.08	0.89 (0.76–1.03)	0.11
Trauma	−0.02	0.05	0.98 (0.89–1.08)	0.68
Tumor	0.14	0.06	1.15 (1.02–1.29)	**0.02**
Cubic age interaction				
Kidney disease	−0.01	0.11	0.99 (0.79–1.24)	0.94
Other unwell	−0.04	0.04	0.97 (0.89–1.05)	0.39
Pruritus	−0.06	0.04	0.94 (0.87–1.02)	0.15
Respiratory	−0.01	0.07	0.99 (0.86–1.13)	0.84
Trauma	−0.03	0.05	0.97 (0.89–1.06)	0.56
Tumor	−0.02	0.05	0.98 (0.88–1.08)	0.64

*n = 40,030/281,543 sick consultations. Random effect variance (± SD): animal 0.55 (0.74), site 0.02 (0.14), practice 0.02 (0.16). Significant (p<0.05) results are displayed in boldface. AVP, Advanced Veterinary Practitioner and/or specialist status; NA, not applicable;OR, odds ratio. RCVS, Royal College of Veterinary Surgeons.

### Cats

#### Prescription of Antimicrobial Drugs

Systemic antimicrobial drugs were prescribed during 32.9% (95% CI 31.9–33.8) of consultations, topical antimicrobials during 6.1% (95% CI 5.9–6.3), and systemic HPCIAs during 17.3% (95% CI 16.2–18.4). The most commonly prescribed class of systemic HPCIA was third-generation cephalosporins (16.4% of consultations for sick cats, 95% CI 15.3–17.6), followed by fluoroquinolones (0.7%, 95% CI 0.4–0.9) and macrolides (0.03%, 95% CI 0.0–0.05). Antimicrobial prescription summarized according to common consultation by breed is shown in Appendix Table 7.

#### Prescription of Systemic Antimicrobial Drugs

Descriptive analyses and univariable model results are summarized in Appendix Table 8. Final multivariable model results are available in Table 5. Odds of systemic antimicrobial prescription were significantly reduced for vaccinated or insured cats. Odds were highest for cats with respiratory and trauma MPCs, although there was a significant interaction between sex and MPC; male cats presented for trauma were significantly more likely than female cats to receive an antimicrobial prescription. Systemic antimicrobial drugs were also less likely to be prescribed for female cats up to ≈15 years of age, when odds for female cats exceeded those for male cats (Figure 1, panel b). Mixed practices were more likely than practices treating companion animals only to prescribe a systemic antimicrobial drug.

**Table 5:** Results from a multivariable mixed effect logistic regression model assessing the association between a range of categorical animal, owner, practitioner and practice-related factors and the probability of prescribing a systemic antimicrobial in cats, United Kingdom, 2014–2016*

Category	β	SE	OR (95% CI)	p value
Intercept				
England	−0.81	0.06	0.45 (0.39–0.50)	NA
Scotland	−0.77	0.10	0.46 (0.38–0.57)	NA
Wales	−0.55	0.12	0.58 (0.46–0.72)	NA
Categorical factors				
Main presenting complaint				
Gastroenteric	NA	NA	Referent	NA
Kidney disease	−0.20	0.07	0.82 (0.71–0.94)	**0.01**
Other unwell	−0.23	0.04	0.79 (0.73–0.85)	**<0.01**
Pruritus	−0.37	0.05	0.69 (0.63–0.76)	**<0.01**
Respiratory	0.91	0.06	2.48 (2.23–2.77)	**<0.01**
Trauma	0.59	0.04	1.80 (1.65–1.97)	**<0.01**
Tumor	−0.56	0.07	0.57 (0.50–0.65)	**<0.01**
Sex				
F	NA	NA	Referent	NA
M	0.03	0.05	1.03 (0.93–1.14)	0.59
Vaccination status				
Not vaccinated	NA	NA	Referent	NA
Vaccinated	−0.09	0.02	0.92 (0.89–0.95)	**<0.01**
Insurance status				
Not insured	NA	NA	Referent	NA
Insured	−0.19	0.02	0.82 (0.79–0.86)	**<0.01**
Genetic breed group (*31*)				
West Europe	NA	NA	Referent	NA
Asian	0.20	0.05	1.22 (1.10–1.36)	**<0.01**
Crossbreed	0.14	0.03	1.16 (1.08–1.23)	**<0.01**
Mediterranean	0.36	0.26	1.43 (0.86–2.38)	0.17
Unclassified	0.11	0.06	1.11 (0.99–1.24)	0.07
Unknown	0.13	0.05	1.14 (1.03–1.26)	**0.01**
Practice type				
Companion animal	NA	NA	Referent	NA
Mixed	0.18	0.08	1.20 (1.03–1.39)	**0.02**
Companion and equine	−0.01	0.18	1.00 (0.70–1.41)	0.98
Companion and large	0.10	0.17	1.10 (0.80–1.53)	0.56
Referral interest				
No	NA	NA	Referent	NA
Yes	−0.08	0.06	0.92 (0.82–1.04)	0.18
Employed RCVS AVP†				
None	NA	NA	Referent	NA
>1 AVP	−0.10	0.07	0.90 (0.79–1.04)	0.16
Continuous factors				
Age				
Linear	−0.38	0.02	0.69 (0.66–0.72)	**<0.01**
Quadratic	−0.08	0.01	0.90 (0.90–0.95)	**<0.01**
Cubic	0.10	0.01	1.08 (1.08–1.12)	**<0.01**
Cats per km^2^ (*30*)				
Linear	−0.02	0.01	0.98 (0.97–1.00)	**0.02**
Interaction terms				
Male sex and age				
Linear age interaction	−0.10	0.03	0.91 (0.85–0.97)	**<0.01**
Quadratic age interaction	−0.10	0.02	0.91 (0.88–0.94)	**<0.01**
Cubic age interaction	0.03	0.02	1.03 (1.00–1.06)	0.11
Male sex and main presenting complaint				
Kidney disease	−0.26	0.11	0.77 (0.62–0.96)	**0.02**
Other unwell	0.17	0.05	1.19 (1.07–1.32)	**<0.01**
Pruritus	0.10	0.07	1.10 (0.96–1.26)	0.16
Respiratory	0.06	0.08	1.06 (0.91–1.23)	0.44
Trauma	0.48	0.06	1.62 (1.44–1.82)	**<0.01**
Tumor	0.15	0.10	1.16 (0.96–1.40)	0.12

*n = 36,521/111,139 sick consultations. Random effect variance (± SD): animal 0.50 (0.70), site 0.06 (0.25), practice 0.08 (0.28). Significant (p<0.05) results are displayed in boldface. NA, not applicable; OR, odds ratio;  RCVS, Royal College of Veterinary Surgeons. †RCVS Advanced Veterinary Practitioner and/or specialist status.

#### Prescription of Systemic HPCIA Drugs

Descriptive analyses and univariable model results are summarized in Appendix Table 9. Final multivariable model results are available in Table 6. Systemic HPCIA drugs were less likely to be prescribed for vaccinated or insured cats. Although odds of prescription were greatest for cats with a respiratory MPC, RCVS-accredited practices were associated with increased odds for cats presented for trauma. Probability of prescription increased for cats up to 6–9 years of age, reduced until ≈18 years of age, and increased again thereafter; prescription was more likely for male cats 5–14 years of age (Figure 2, panel B). In terms of genetic breed, the greatest odds of systemic HPCIA prescription were for the Asian group compared with the West Europe group.

**Table 6:** Results from a multivariable mixed effect logistic regression model assessing the association between a range of categorical animal, owner, practitioner and practice-related factors and the probability of prescribing a systemic highest priority critically important antimicrobial drug for cats, United Kingdom, 2014–2016*

Category	β	SE	OR (95% CI)	p value
Intercept				
England	−2.79	0.21	0.06 (0.04–0.09)	NA
Scotland	−2.74	0.24	0.07 (0.04–0.10)	NA
Wales	−2.55	0.24	0.08 (0.05–0.12)	NA
Categorical factors				
Main presenting complaint	NA	NA	Referent	NA
Gastroenteric	0.55	0.25	1.74 (1.08–2.82)	**0.02**
Kidney disease	0.59	0.12	1.80 (1.43–2.26)	**<0.01**
Other unwell	1.08	0.13	2.95 (2.28–3.81)	**<0.01**
Pruritus	1.50	0.14	4.47 (3.41–5.85)	**<0.01**
Respiratory	1.06	0.12	2.89 (2.27–3.67)	**<0.01**
Trauma	0.38	0.18	1.46 (1.04–2.03)	**0.03**
Tumor	NA	NA	Referent	NA
Sex	0.12	0.03	1.13 (1.07–1.19)	**<0.01**
F	NA	NA	Referent	NA
M	−0.06	0.02	0.95 (0.91–0.98)	**<0.01**
Vaccination status	NA	NA	Referent	NA
Not vaccinated	−0.14	0.03	0.87 (0.83–0.92)	**<0.01**
Vaccinated	NA	NA	Referent	NA
Insurance status	0.05	0.03	1.05 (1.00–1.11)	0.06
Not insured	NA	NA	Referent	NA
Insured	0.21	0.07	1.23 (1.08–1.40)	**<0.01**
Genetic breed group (*31*)	0.14	0.04	1.16 (1.06–1.26)	**<0.01**
West Europe	0.11	0.32	1.12 (0.59–2.11)	0.73
Asian	0.14	0.07	1.15 (1.00–1.33)	0.06
Crossbreed	0.12	0.06	1.12 (0.99–1.27)	0.07
Accreditation status				
Not accredited	NA	NA	Referent	NA
>1 accredited site	0.10	0.22	1.10 (0.72–1.69)	0.65
Continuous factors				
Age				
Linear	−0.23	0.03	0.80 (0.76–0.85)	**<0.01**
Quadratic	−0.13	0.02	0.88 (0.85–0.90)	**<0.01**
Cubic	0.13	0.01	1.14 (1.11–1.17)	**<0.01**
Interaction terms				
Main presenting complaint and accreditation (accredited site)				
Kidney disease	0.23	0.26	1.26 (0.76–2.08)	0.37
Other unwell	0.21	0.13	1.23 (0.96–1.58)	0.10
Pruritus	0.00	0.14	1.00 (0.76–1.32)	1.00
Respiratory	0.23	0.15	1.26 (0.94–1.69)	0.12
Trauma	0.64	0.13	1.90 (1.46–2.47)	**<0.01**
Tumor	0.19	0.19	1.21 (0.83–1.75)	0.32
Male sex and age				
Linear age interaction	−0.06	0.04	0.95 (0.87–1.02)	0.17
Quadratic age interaction	−0.09	0.02	0.91 (0.87–0.95)	**<0.01**
Cubic age interaction	0.02	0.02	1.02 (0.98–1.06)	0.32

*n = 19,018/111,139 sick consultations. Random effect variance (± SD): animal 0.68 (0.82, site 0.13 (0.36), practice 0.44 (0.66). Significant (p<0.05) results are displayed in boldface. NA, not applicable; OR, odds ratio.

#### Prescription of Topical Antimicrobial Drugs

Descriptive analyses and univariable model results are summarized in Appendix Table 10. Final multivariable model results are available in Table 7. Topical antimicrobial drugs were less likely to be prescribed for insured cats. The effect of age at consultation varied according to MPC; probability of prescription decreased for pruritic cats until ≈7 years of age, before increasing again (Figure 3, panel B). In terms of genetic breed, odds were smallest for crossbreeds compared with those in the West Europe genetic breed group.

**Table 7:** Results from a multivariable mixed effect logistic regression model assessing the association between a range of categorical animal, owner, practitioner and practice-related factors and the probability of prescribing a topical antimicrobial in cats, United Kingdom, 2014–2016*

Category	β	SE	OR (95% CI)	p value
Intercept				
England	−3.98	0.17	0.02 (0.01–0.03)	-NA
Scotland	−3.94	0.19	0.02 (0.01–0.03)	NA
Wales	−3.91	0.19	0.02 (0.01–0.03)	NA
Categorical factors				
Main presenting complaint				
Gastroenteric	NA	NA	Referent	NA
Kidney disease	−0.98	0.50	0.38 (0.14–1.00)	0.05
Other unwell	1.79	0.16	5.96 (4.37–8.12)	**<0.01**
Pruritus	2.13	0.16	8.37 (6.09–11.51)	**<0.01**
Respiratory	1.21	0.18	3.36 (2.35–4.82)	**<0.01**
Trauma	1.34	0.17	3.82 (2.76–5.28)	**<0.01**
Tumor	0.38	0.25	1.46 (0.90–2.36)	0.12
Sex				
F	NA	NA	Referent	NA
M	0.05	0.03	1.05 (1.00–1.11)	0.06
Neutered status				
Not neutered	NA	NA	Referent	NA
Neutered	−0.06	0.04	0.94 (0.88–1.01)	0.09
Insurance status				
Not insured	NA	NA	Referent	NA
Insured	−0.13	0.04	0.88 (0.82–0.95)	**<0.01**
Genetic breed group (*31*)				
West Europe	NA	NA	Referent	NA
Asian	−0.14	0.09	0.87 (0.73–1.03)	0.09
Crossbreed	−0.50	0.05	0.61 (0.55–0.67)	**<0.01**
Mediterranean	−0.40	0.50	0.67 (0.25–1.78)	0.42
Unclassified	−0.24	0.09	0.79 (0.66–0.95)	**0.01**
Unknown	−0.43	0.08	0.65 (0.56–0.77)	**<0.01**
Referral interest				
No	NA	NA	Referent	NA
Yes	0.08	0.05	1.08 (0.98–1.19)	0.11
Continuous factors				
Age				
Linear	0.08	0.26	1.09 (0.65–1.82)	0.75
Quadratic	−0.12	0.14	0.89 (0.68–1.17)	0.40
Cubic	−0.14	0.14	0.87 (0.66–1.15)	0.34
Interaction terms				
Main presenting complaint and age				
Linear age interaction				
Kidney disease	1.14	0.68	3.11 (0.82–11.84)	0.10
Other unwell	−0.61	0.27	0.54 (0.32–0.91)	**0.02**
Pruritus	0.18	0.27	1.19 (0.70–2.03)	0.52
Respiratory	−0.34	0.31	0.71 (0.39–1.29)	0.26
Trauma	0.07	0.28	1.07 (0.62–1.85)	0.81
Tumor	−0.07	0.38	0.93 (0.44–1.95)	0.85
Quadratic age interaction				
Kidney disease	0.52	0.32	1.69 (0.89–3.18)	0.11
Other unwell	0.16	0.14	1.17 (0.89–1.53)	0.26
Pruritus	0.42	0.14	1.52 (1.15–2.02)	**<0.01**
Respiratory	0.26	0.16	1.29 (0.95–1.77)	0.11
Trauma	0.22	0.15	1.24 (0.93–1.65)	0.14
Tumor	0.16	0.20	1.18 (0.80–1.73)	0.41
Cubic age interaction				
Kidney disease	−0.51	0.33	0.60 (0.31–1.16)	0.13
Other unwell	0.14	0.14	1.15 (0.87–1.52)	0.33
Pruritus	0.04	0.15	1.04 (0.78–1.38)	0.81
Respiratory	−0.03	0.16	0.97 (0.70–1.33)	0.84
Trauma	0.06	0.15	1.06 (0.79–1.42)	0.70
Tumor	0.10	0.19	1.10 (0.75–1.61)	0.62

*n = 6,769/111,139 sick consultations. Random effect variance (± SD): animal 0.82 (0.90,) site 0.02 (0.15), practice 0.03 (0.16). Significant (p<0.05) results are displayed in boldface. OR, odds ratio; RCVS, Royal College of Veterinary Surgeons.

## Discussion

We demonstrated frequent prescription of antimicrobial drugs, including systemic HPCIAs (particularly in cats), in veterinary practices in Great Britain. Considering the importance of HPCIAs in the context of antimicrobial resistance (*32*), we identified a vital need to learn more about factors potentially driving such prescribing behaviors. We further augmented EHR data with a range of external data sources to identify key client, animal, and practice-related risk factors associated with prescription of systemic and topical antimicrobial and systemic HPCIA drugs; such factors potentially inform key antimicrobial stewardship targets of importance to companion animal practice.

Regarding client care decision-related factors, odds of prescription for systemic antimicrobial and HPCIA drugs were significantly lower for vaccinated dogs and cats, possibly reflecting perceived or actual reduced risk for antimicrobial drug–responsive disease in vaccinated animals. Although most companion animal vaccines target viruses, bacterial infection secondary to vaccine-preventable viral disease has been documented (*33*). Risk avoidance plays a major role in antimicrobial drug–prescribing practices (*12*), potentially prompting more frequent prescription for unwell, nonvaccinated animals. We speculate that previous engagement with preventive healthcare services might select for clients more likely to seek veterinary attention earlier or to pursue diagnostic options rather than empirical prescription. Regardless of what might be driving these trends, the O’Neill report recommends that promoting development and use of vaccines and alternatives to antibiotics should form a key component of efforts to curtail human dissemination of antimicrobial resistance (*34*); our findings suggest that such recommendations should also be considered for companion animals.

Insurance coverage was associated with decreased odds of prescription of systemic and topical antimicrobial drugs, potentially highlighting veterinary practitioners being more likely to seek a wider range of diagnostic options in preference to empirical antibiosis for insured animals. However, insured dogs were also associated with increased odds of prescription of systemic HPCIA drugs. Cost of therapy has been shown to influence choice of antimicrobial agent for companion animals (*17*), and HPCIA drugs are anecdotally considered a more expensive option than other antimicrobial drugs. Hence, our findings might reflect increased willingness to prescribe relatively expensive antimicrobial drugs for insured dogs.

Although HPCIA drug classification remains under debate, use of HPCIA drugs has formed a focus for antimicrobial resistance–related policy (*34*). Although several classes of HPCIA drugs (e.g., glycopeptides, which are not authorized for use in animals) are very rarely prescribed for companion animals in the United Kingdom (*22*), prescription of fluoroquinolones and third-generation cephalosporins (particularly for cats) is relatively common, although current antimicrobial drug prescribing guidance strongly discourages such practices (*35*).

With regard to animal-intrinsic factors, odds for prescription of systemic antimicrobial drugs were increased for younger male cats, although the opposite was found for dogs. Sex-based variation in risk for bacterial infection has been identified (*36*–*38*), and cat fight–related injuries are a frequently recorded clinical complaint (*39*), more commonly associated with young outdoor-ranging male cats (*40*). Indeed, we found that systemic antimicrobial drugs were more commonly prescribed for male cats presented for trauma. Furthermore, time of injury is less likely to be known for outdoor-ranging cats than for dogs; such uncertainty might prompt a more cautious approach to prescription of antimicrobial drugs (*41*).

Other studies have also identified age- or sex-related variation in risk for antimicrobial resistance (*36*–*38*). For instance, Radford et al. demonstrated decreased probability of systemic antimicrobial prescription with increased patient age (*20*), potentially reflecting increased actual or perceived incidence of noncommunicable disease as animals age. This interpretation might partly explain our findings, although we noted an exception with prescription of systemic HPCIA drugs. For cats, an easy-to-administer (injectable) long-acting third-generation cephalosporin formulation is widely used (*21*–*23*). Although not completely explanatory, our findings may suggest that as an animal ages, the client or veterinarian perceives an increased probability of an animal being refractory to an intervention (e.g., oral administration of tablets), increasing the likelihood of a prescriber choosing easy-to-administer formulations. A previously identified key influencer of antimicrobial agent choice was administration of inappropriate dosages as a result of noncompliance (*17*). Whether the risk for antimicrobial resistance posed by a possible underdose of a first-line antimicrobial outweighs the risk posed by the labeled dose of a third-line HPCIA drug remains unanswered.

As with humans (*10*,*11*,*13*), prescription of systemic antimicrobial drugs for dogs and cats was most commonly associated with respiratory clinical signs. Humans having respiratory conditions are often inappropriately prescribed antimicrobial drugs; most respiratory conditions are viral or noninfectious in origin (*10*). The same has also been shown for companion animals, although bacterial infection secondary to primary viral disease has been documented (*42*). Considering these shared patterns, although prescribing guidance is available (*43*), we suggest respiratory disease as a pertinent area for further investigation of One Health antimicrobial stewardship intervention methods.

Increased odds of prescription of topical antimicrobial drugs was commonly associated with the retriever group of dogs, which contains several breeds commonly associated with dermatologic disease (*44*). This finding and interpretation is plausible, suggesting that the breed summarization technique used here to combat the modeling issues posed by the >250 dog and >50 cat breeds recorded in this dataset was useful. However, genetic linkage does not necessarily imply phenotypic similarity. As such, individual breed-level phenotypes might be responsible for conferring variant bacterial infection risk in ways not explored, and indeed potentially masked. In future analyses, we will aim to identify additional means by which breeds can be effectively summarized according to shared genotype and phenotype.

Although the individual animal accounted for most of the random effect variance seen in this study, veterinary-led factors might well yield more readily accessible routes toward stewardship. For site accreditation, the voluntary RCVS Practice Standards Scheme requires antimicrobial drug use policies, infection control plans, and established clinical audits (*45*), and we observed reduced systemic antimicrobial prescription odds for dogs in accredited practices. Although practices seeking accreditation might already be more engaged with quality improvement, we nevertheless recommend further consideration as to whether the RCVS Practice Standards Scheme could play a more central role for encouraging stewardship in general and referral practices.

Compared with practices that treat companion animals only, mixed species practices were associated with increased odds of prescription of systemic antimicrobial drugs. Veterinary surgeons employed in different sectors expressed varied attitudes toward antimicrobial resistance (*16*), a finding perhaps demonstrated on a wide scale in this study. Practices employing RCVS specialists were also associated with reduced odds of prescription of topical antimicrobial drugs for dogs, potentially reflecting varied case management approaches (*46*) or caseloads compared with general practices.

Considering limitations of this study, although we successfully augmented EHRs with a variety of data sources, no dataset is infallible. For instance, the veterinary surgeon employment record of the RCVS Practice Register is updated only on an ad hoc basis. It is thus possible that the surveyed veterinary surgeon population varied over the 2-year study period in ways not captured here. Veterinary practices participating in SAVSNET are recruited by convenience and might not be representative of the wider UK population. Although we found no clear associations between IMD or pet population density and prescription, the complexities of summarizing IMD across the constituent countries of the United Kingdom (*47*), coupled with the relative infancy of pet population demographic studies (*30*), lead us to recommend re-evaluation as research methods further mature. The analyzed population was relatively skewed toward less deprived areas; to ascertain whether this finding is reflective of the wider UK pet-owning community, including the charity and low-income veterinary sectors in future analyses would be warranted. We advise caution for inferring causal relationships between factors and outcome variables explored in this cross-sectional study; similarly, group-level observations might have limited relevance to individual animals. More generalized SAVSNET limitations have been previously discussed; in brief, quantification of antimicrobial drug prescription depends on practitioners charging for antimicrobial drugs, and analyzed practices were recruited by convenience (*22*,*30*).

In conclusion, we demonstrated the value of using veterinary EHRs collected from a cohort of veterinary practices to identify a range of factors associated with prescription of antimicrobial drugs for dogs and cats. Although factors influencing decision-making remain multifactorial and complex, our findings suggest that gathering clinical evidence surrounding respiratory disease might be of value to stewardship. Preventive healthcare could also play a role in stewardship and should form the basis of client-targeted health messaging, as should the RCVS Practice Standards Scheme for veterinary practitioners.

Appendix Supplemental results for study of factors associated with prescription of antimicrobial drugs for dogs and cats, United Kingdom, 2014–2016.
