# Supplementary material for: Factors Associated with Prescription of Antimicrobial Drugs for Dogs and Cats, United Kingdom, 2014–2016
Source: Emerg Infect Dis. 2020 Aug;26(8):1778–91. doi: 10.3201/eid2608.191786 (PMC7392421; doi:10.3201/eid2608.191786)
Supplement: Appendix — Supplemental results for study of factors associated with prescription of antimicrobial drugs for dogs and cats, United Kingdom, 2014–2016. [file 19-1786-Techapp-s1.pdf]

Article DOI: https://doi.org/10.3201/eid2608.191786

Factors Associated with Prescription of Antimicrobial Drugs for Dogs and Cats, United Kingdom, 2014–2016

Appendix

Appendix Table 1. Summary of antimicrobial agents authorised for use in dogs and/or cats in the United Kingdom. Information source: Veterinary Medicines Directorate (https://www.vmd.defra.gov.uk/ProductInformationDatabase/), accessed 1 April 2016.

Antimicrobial class	Antimicrobial agent
Aminoglycoside	Framycetin sulfate
	Gentamicin
	Neomycin
	Streptomycin sulfate
Amphenicol	Florfenicol
Beta-lactam	
Amoxicillin	Amoxicillin
Ampicillin	Ampicillin
Clavulanic acid potentiated amoxicillin	Amoxicillin-clavulanic acid
Cloxacillin	Cloxacillin
1st generation cephalosporin	Cefalexin
3rd generation cephalosporin	Cefovecin
Penicillin	Benzathine benzyl penicillin
Penicillin	Procaine benzylpenicillin
Fluoroquinolone	Enrofloxacin
	Orbifloxacin
	Marbofloxacin
	Pradofloxacin
Fusidic acid	Fusidic acid
Lincosamide	Clindamycin
	Lincomycin
Nitroimidazole	Metronidazole
Nitroimidazole-macrolide	Metronidazole-spiramycin
Potentiated sulphonamide	Sulfadiazine-trimethoprim
Polymyxin	Polymyxin B sulfate
Tetracycline	Doxycycline
	Oxytetracycline

Appendix Table 2. Descriptive demographic summary of sick canine and feline consultations used for analyses of factors associated with antimicrobial prescription, focusing on the percentage of consultations contributed by a range of genetically similar breed groups, as defined by Vonholdt et al. (2010) for dog breeds, and Lipinski et al. (2008) for cat breeds.

Breeds	% of consults (95% CI)	Breeds	% of consults (95% CI)
Dog breed group		Cat breed group	
Ancient / spitz	1.3 (1.2–1.4)	Asian	3.5 (3.3–3.8)
Crossbreed	22.1 (21.4–22.8)	Crossbreed	87.6 (86.3–88.8)
Herding	4.7 (4.4–5.1)	Mediterranean	0.1 (0.1–0.1)
Mastiff-like	9.5 (9.1–9.9)	West Europe	6.4 (5.3–7.5)
Retriever	14.5 (13.8–15.2)	Unclassified	2.5 (2.3–2.7)
Scent hound	2.6 (2.5–2.8)	Unknown / missing	4.0 (3.1–4.8)
Sight hound	1.6 (1.5–1.8)		
Small terriers	12.8 (12.4–13.2)		
Spaniel	13.7 (13.3–14.1)		
Toy	4.7 (4.4–5.0)		
Working dog	5.2 (5.0–5.4)		
Unclassified	11.3 (10.9–11.6)		
Unknown / missing	1.2 (1.0–1.4)		

Appendix Table 3. Descriptive summary of the percentage of total sick canine consultations where an animal was prescribed at least one antimicrobial (systemic, topical or systemic highest priority critically important (HPCIA) compared against animal breed, including breeds where in excess of 2,500 consultations were recorded*

Genetic breed group (*1*)	Dog breed	*n* consults	Systemic %†	Systemic 95% CI	Topical %	Topical 95% CI	Systemic HPCIA %	Systemic HPCIA 95% CI
Crossbreed	Crossbreed	59,010	24.9	23.9–25.8	13.3	12.9–13.7	1.2	0.9–1.4
Herding	Border collie	9,821	26.7	25.2–28.2	8.1	7.5–8.7	1.0	0.6–1.5
	Border terrier	5,225	24.3	22.6–26.1	16.0	14.7–17.3	1.4	0.9–1.8
Mastiff-like	Boxer	4,780	22.6	21.0–24.2	17.7	16.4–19.1	0.7	0.4–0.9
	Bulldog	2,530	32.7	30.5–34.9	23.3	21.3–25.3	1.1	0.6–1.6
	Staffordshire bull terrier	9,719	24.8	23.6–26.0	15.6	14.8–16.5	0.7	0.5–1.0
Retriever	Golden retriever	6,223	26.3	24.4–28.1	15.1	13.9–16.4	1.0	0.7–1.4
	Labrador retriever	30,977	22.7	21.6–23.8	15.2	14.5–15.9	1.0	0.7–1.2
Scent hound	Dachshund	3,065	25.1	22.7–27.4	9.6	8.4–10.9	2.7	1.8–3.5
Small terrier	Jack russell terrier	14,869	26.1	24.9–27.4	16.7	15.8–17.7	1.4	1.1–1.8
	West highland white terrier	11,040	28.9	27.5–30.3	10.8	10.0–11.7	2.9	2.4–3.5
	Yorkshire terrier	6,328	27.6	25.9–29.2	11.0	10.4–11.6	3.2	2.6–3.8
Spaniel	Cavalier King Charles spaniel	7,586	22.5	21.1–24.0	14.0	13.1–14.9	1.3	0.9–1.7
	Cocker spaniel	15,312	27.8	26.5–29.2	18.1	17.2–18.9	1.7	1.4–2.1
	English springer spaniel	6,774	26.3	24.8–27.9	14.1	13.1–15.2	1.3	0.9–1.7
	Springer spaniel	4,073	27.4	25.6–29.2	15.5	14.1–16.8	1.4	0.9–1.9
Toy	Chihuahua	2,583	26.5	24.3–28.8	7.9	6.8–9.0	2.3	1.5–3.1
	Pug	2,679	24.7	22.6–26.7	21.5	19.9–23.1	1.8	1.1–2.4
	Shih tzu	5,938	23.4	21.8–25.0	17.3	16.2–18.5	2.0	1.6–2.5
Unclassified	Bichon frise	3,314	25.8	24.2–27.4	18.7	17.1–20.4	1.4	0.9–1.8
	Lhasa apso	3,060	26.5	24.3–28.7	17.3	15.5–19.1	2.4	1.7–3.1
Unknown	Unknown	3,182	24.3	22.5–26.1	12.1	10.8–13.3	0.9	0.5–1.3
Working dog	German shepherd dog	6,695	28.4	27.0–29.8	13.5	12.5–14.4	1.1	0.7–1.6
	Schnauzer	3,376	27.2	25.2–29.1	12.3	11.0–13.5	1.2	0.7–1.8

*HPCIA, Highest priority critically important antimicrobial.

Appendix Table 4. Descriptive summary of the percentage of total sick canine consultations prescribed a systemic antimicrobial. Also included are parameter estimates from a series of univariable mixed effect logistic regression models assessing the association between a range of animal, owner, practitioner and practice-related factors and the probability of prescribing a systemic antimicrobial. Random effects include animal, site, and practice*

Variable	Category	% of prescribing consults (95% CI)	β	SE	OR	95% CI	*P*
Categorical factors							
Country	England (Intercept)	25.7 (24.7–26.7)	–1.16	0.03	0.31	0.30–0.33	
	Scotland	26.8 (24.9–28.7)	0.04	0.05	1.04	0.94–1.16	0.45
	Wales	24.7 (22.3–27.1)	–0.02	0.07	0.98	0.86–1.12	0.76
Main presenting complaint	Gastroenteric (Intercept)	40.2 (41.0–44.8)	–0.46	0.03	0.63	0.59–0.67	
	Other unwell	22.0 (21.3–22.8)	–0.93	0.02	0.34	0.38–0.41	<0.01
	Kidney disease	30.1 (27.4–32.8)	–0.39	0.06	0.68	0.61–0.76	<0.01
	Pruritus	27.0 (25.7–28.4)	–0.65	0.02	0.52	0.51–0.54	<0.01
	Respiratory	42.9 (41.0–44.8)	0.11	0.03	1.12	1.06–1.17	<0.01
	Trauma	22.5 (21.5–23.6)	–0.86	0.02	0.42	0.41–0.44	<0.01
	Tumour	18.4 (17.5–19.3)	–1.17	0.03	0.31	0.30–0.33	<0.01
Sex	Female (Intercept)	25.9 (24.9–26.8)	–1.15	0.03	0.32	0.30–0.33	
	Male	25.6 (24.7–26.4)	–0.01	0.01	0.99	0.97–1.01	0.19
Neuter status	Un-neutered (Intercept)	27.4 (26.5–28.2)	–1.08	0.03	0.34	0.32–0.36	
	Neutered	24.8 (24.0–25.7)	–0.12	0.01	0.89	0.87–0.91	<0.01
Microchip status	Un-microchipped (Intercept)	26.4 (25.5–27.3)	–1.14	0.03	0.32	0.30–0.34	
	Microchipped	25.2 (24.3–26.1)	–0.03	0.01	0.97	0.95–0.99	0.01
Vaccination status	Un-vaccinated (Intercept)	27.3 (26.4–28.2)	–1.10	0.03	0.33	0.32–0.35	
	Vaccinated	25.1 (24.2–26.0)	–0.09	0.01	0.92	0.90–0.94	<0.01
Insurance status	Un-insured (Intercept)	26.7 (25.9–27.6)	–1.11	0.03	0.33	0.31–0.35	
	Insured	23.7 (22.7–24.7)	–0.14	0.01	0.87	0.85–0.89	<0.01
Owner urban status	Urban (Intercept)	25.5 (24.5–26.4)	–1.16	0.03	0.31	0.30–0.33	
	Rural	26.2 (25.0–27.3)	0.01	0.01	1.01	0.98–1.03	0.71
Genetic breed group (*1*)	Retriever (Intercept)	23.4 (22.3–24.5)	–1.28	0.03	0.28	0.26–0.29	
	Crossbreed	24.9 (23.9–25.8)	0.08	0.02	1.08	1.05–1.12	<0.01
	Ancient / spitz	28.8 (26.7–30.8)	0.27	0.05	1.32	1.20–1.44	<0.01

Variable	Category	% of prescribing consults (95% CI)	β	SE	OR	95% CI	*P*
	Herding	26.5 (25.2–27.8)	0.14	0.03	1.15	1.09–1.22	<0.01
	Mastiff-like	26.2 (25.2–27.1)	0.16	0.02	1.17	1.12–1.22	<0.01
	Scent hound	25.6 (24.0–27.1)	0.13	0.04	1.13	1.06–1.21	<0.01
	Sight hound	29.5 (27.6–31.5)	0.30	0.04	1.35	1.25–1.47	<0.01
	Small terrier	27.3 (26.2–28.4)	0.20	0.02	1.22	1.17–1.27	<0.01
	Spaniel	26.5 (25.4–27.5)	0.16	0.02	1.17	1.13–1.22	<0.01
	Toy	24.7 (23.4–25.9)	0.06	0.03	1.06	1.01–1.12	0.03
	Unclassified	26.0 (25.0–27.0)	0.13	0.02	1.14	1.09–1.19	<0.01
	Unknown	24.3 (22.6–26.1)	0.12	0.05	1.13	1.03–1.24	0.01
	Working dog	27.4 (26.4–28.4)	0.21	0.03	1.24	1.18–1.30	<0.01
Practice type	Small animal (Intercept)	25.4 (24.3–26.4)	−1.19	0.03	0.31	0.29–0.32	
	Mixed	26.6 (25.0–28.3)	0.16	0.07	1.18	1.03–1.34	0.02
	Small & equine	23.1 (20.2–25.9)	−0.04	0.15	0.96	0.71–1.30	0.79
	Small & large	28.7 (26.2–31.2)	0.16	0.14	1.17	0.89–1.55	0.27
Accreditation	Not accredited (Intercept)	28.4 (26.3–30.5)	−0.93	0.07	0.40	0.35–0.46	
	1+ accredited site	25.2 (24.3–26.1)	−0.27	0.07	0.77	0.66–0.89	<0.01
Hospital status	No hospital site (Intercept)	26.2 (25.2–27.2)	−1.14	0.03	0.32	0.30–0.34	
	1+ hospital site	23.9 (22.7–25.1)	−0.09	0.06	0.91	0.81–1.04	0.16
Referral interest	No (Intercept)	26.0 (25.1–26.9)	−1.12	0.03	0.33	0.31–0.35	
	Yes	25.1 (23.2–26.9)	−0.11	0.05	0.89	0.80–0.99	0.04
Employed RCVS AVP†	None (Intercept)	26.3 (25.3–27.2)	−1.13	0.03	0.32	0.31–0.34	
	1+ AVP	24.0 (22.2–25.8)	−0.14	0.06	0.87	0.77–0.98	0.02
Employed RCVS	None (Intercept)	25.8 (25.0–26.7)	−1.15	0.03	0.32	0.30–0.33	
specialist†	1+ specialist	22.0 (19.1–24.8)	−0.18	0.15	0.84	0.63–1.11	0.21
Continuous factors							
Age (years)	Intercept		−1.14	0.03	0.32	0.31–0.34	
	Age - linear		−0.10	0.01	0.90	0.88–0.92	<0.01
	Age - quadratic		−0.03	0.01	0.97	0.96–0.99	<0.01
	Age - cubic		0.02	0.01	1.02	1.02–1.03	<0.01
rIMD‡	Intercept		−1.16	0.03	0.31	0.30–0.33	
	rIMD		−0.02	0.01	0.98	0.97–1.00	0.04
Dogs per household (*2*)	Intercept		−1.16	0.03	0.31	0.30–0.33	
	Dogs per household		−0.01	0.01	0.99	0.98–1.01	0.24
Dogs per km^2 (*2*)	Intercept		−1.16	0.03	0.31	0.30–0.33	
	Dogs per km		−0.01	0.01	1.00	0.98–1.01	0.34

*SE, standard error; OR, odds ratio.
†Royal College of Veterinary Surgeons (RCVS) Advanced Veterinary Practitioner (AVP) and / or specialist status.
‡Rescaled Indices of Multiple Deprivation (rIMD) quintile, 1 = most deprived.

Appendix Table 5. Descriptive summary of the percentage of total sick canine consultations prescribed a systemic highest priority critically important antimicrobial (HPCIA). Also included are parameter estimates from a series of univariable mixed effect logistic regression models assessing the association between a range of animal, owner, practitioner and practice-related factors and the probability of prescribing a systemic HPCIA. Random effects include animal, site, and practice*

Variable	Category	% of prescribing consults (95% CI)	β	SE	OR	95% CI	*P*
Categorical factors							
Country	England (Intercept)	1.4 (1.2–1.7)	–4.80	0.07	0.01	0.01–0.01	1
	Scotland	1.4 (0.9–1.8)	–0.15	0.19	0.86	0.59–1.24	0.42
	Wales	1.1 (0.7–1.6)	–0.11	0.20	0.90	0.61–1.32	0.59
Main presenting complaint	Gastroenteric (Intercept)	1.7 (0.8–2.7)	–4.54	0.08	0.01	0.01–0.01	
	Kidney disease	2.2 (1.5–2.8)	0.31	0.18	1.36	0.95–1.95	0.09
	Other unwell	1.5 (1.3–1.8)	–0.21	0.06	0.81	0.73–0.91	<0.01
	Pruritus	1.6 (1.3–1.8)	–0.18	0.07	0.84	0.74–0.95	<0.01
	Respiratory	2.8 (2.4–3.3)	0.44	0.08	1.55	1.31–1.82	<0.01
	Trauma	0.5 (0.4–0.7)	–1.13	0.08	0.32	0.27–0.38	<0.01
	Tumour	0.8 (0.6–1.0)	–0.80	0.11	0.45	0.37–0.56	<0.01
Sex	Female (Intercept)	1.4 (1.2–1.7)	–4.80	0.07	0.01	0.01–0.01	
	Male	1.4 (1.2–1.6)	–0.03	0.04	0.97	0.90–1.05	0.47
Neuter status	Un-neutered (Intercept)	1.4 (1.2–1.6)	–4.82	0.07	0.01	0.01–0.01	
	Neutered	1.4 (1.2–1.7)	0.00	0.04	1.00	0.92–1.09	0.94
Microchip status	Un-microchipped (Intercept)	1.5 (1.3–1.7)	–4.75	0.07	0.01	0.01–0.01	
	Microchipped	1.4 (1.1–1.6)	–0.12	0.04	0.88	0.82–0.96	<0.01
Vaccination status	Un-vaccinated (Intercept)	1.5 (1.3–1.7)	–4.73	0.07	0.01	0.01–0.01	
	Vaccinated	1.4 (1.2–1.6)	–0.13	0.04	0.88	0.81–0.96	<0.01
Insurance status	Un-insured (Intercept)	1.3 (1.1–1.6)	–4.86	0.07	0.01	0.01–0.01	
	Insured	1.5 (1.3–1.8)	0.13	0.04	1.13	1.04–1.23	<0.01
Owner urban status	Urban (Intercept)	1.3 (1.1–1.5)	–4.83	0.07	0.01	0.01–0.01	
	Rural	1.6 (1.3–2.0)	0.03	0.05	1.04	0.95–1.14	0.49
Genetic breed group (*1*)	Retriever (Intercept)	1.0 (0.7–1.2)	–5.19	0.09	0.01	0.01–0.01	
	Crossbreed	1.2 (0.9–1.4)	0.06	0.22	1.07	0.69–1.64	0.78
	Ancient / spitz	0.9 (0.5–1.3)	0.24	0.08	1.27	1.09–1.47	<0.01
	Herding	1.2 (0.7–1.6)	0.08	0.12	1.09	0.86–1.37	0.50
	Mastiff-like	1.0 (0.8–1.1)	0.09	0.10	1.09	0.90–1.33	0.37
	Scent hound	1.9 (1.4–2.4)	0.67	0.13	1.95	1.52–2.51	<0.01
	Sight hound	1.4 (0.9–1.8)	0.34	0.17	1.41	1.01–1.97	0.04
	Small terrier	2.3 (1.9–2.6)	0.80	0.08	2.23	1.91–2.61	<0.01
	Spaniel	1.5 (1.3–1.8)	0.45	0.08	1.58	1.34–1.80	<0.01
	Toy	2.2 (1.8–2.6)	0.90	0.10	2.45	2.02–2.99	<0.01
	Unclassified	1.5 (1.2–1.8)	0.43	0.09	1.53	1.29–1.81	<0.01
	Unknown	0.9 (0.5–1.3)	0.18	0.22	1.20	0.77–1.85	0.43
	Working dog	1.4 (1.1–1.8)	0.45	0.11	1.57	1.27–1.93	<0.01
Practice type	Small animal (Intercept)	1.3 (1.1–1.5)	–4.85	0.07	0.01	0.01–0.01	
	Mixed	1.7 (1.0–2.3)	0.18	0.17	1.20	0.86–1.66	0.29
	Small & equine	1.2 (0.7–1.6)	–0.10	0.40	0.91	0.42–1.98	0.80
	Small & large	1.5 (1.0–1.9)	0.08	0.35	1.09	0.55–2.15	0.81
Accreditation	Not accredited (Intercept)	1.7 (1.1–2.4)	–4.65	0.18	0.01	0.01–0.01	
	1+ accredited site	1.4 (1.1–1.6)	–0.19	0.19	0.83	0.57–1.20	0.33
Hospital status	No hospital site (Intercept)	1.5 (1.3–1.8)	–4.78	0.07	0.01	0.01–0.01	
	1+ hospital site	1.0 (0.9–1.1)	–0.17	0.16	0.84	0.62–1.15	0.28
Referral interest	No (Intercept)	1.5 (1.2–1.7)	–4.80	0.08	0.01	0.01–0.01	
	Yes	1.2 (1.0–1.5)	–0.06	0.14	0.94	0.72–1.23	0.66
Employed RCVS AVP†	None (Intercept)	1.5 (1.2–1.7)	–4.79	0.07	0.01	0.01–0.01	
	1+ AVP	1.3 (1.0–1.5)	–0.13	0.16	0.87	0.64–1.19	0.39
Employed RCVS specialist†	None (Intercept)	1.4 (1.2–1.6)	–4.81	0.06	0.01	0.01–0.01	
	1+ specialist	0.8 (0.5–1.1)	–0.26	0.38	0.77	0.37–1.62	0.49
		Continuous factors					
Age (years)	Intercept		–4.81	0.07	0.01	0.01–0.01	
	Age - linear		0.20	0.04	1.22	1.13–1.32	<0.01
	Age - quadratic		–0.03	0.03	0.97	0.93–1.02	0.23
	Age - cubic		0.04	0.02	1.04	1.01–1.08	0.01
rIMD‡	Intercept		–4.82	0.06	0.01	0.01–0.01	
	rIMD		0.02	0.02	1.02	0.97–1.07	0.39
Dogs per household (*2*)	Intercept		–4.82	0.06	0.01	0.01–0.01	
	Dogs per household		0.02	0.03	1.03	0.97–1.09	0.40
Dogs per km^2 (*2*)	Intercept		–4.82	0.06	0.01	0.01–0.01	
	Dogs per km		–0.02	0.02	0.98	0.94–1.02	0.31

*SE, standard error; OR, odds ratio.
†Royal College of Veterinary Surgeons (RCVS) Advanced Veterinary Practitioner (AVP) and / or specialist status.
‡Rescaled Indices of Multiple Deprivation (rIMD) quintile, 1 = most deprived.

Appendix Table 6. Descriptive summary of the percentage of total sick canine consultations prescribed a topical antimicrobial. Also included are parameter estimates from a series of univariable mixed effect logistic regression models assessing the association between a range of animal, owner, practitioner and practice-related factors and the probability of prescribing a topical antimicrobial. Random effects include animal, site, and practice*

Variable	Category	% of prescribing consults (95% CI)	β	SE	OR	95% CI	*P*
Categorical factors							
Country	England (Intercept)	14.1 (13.9–14.6)	–1.82	0.02	0.16	0.16–0.17	
	Scotland	13.4 (11.9–14.9)	0.03	0.05	1.03	0.93–1.13	0.58
	Wales	14.7 (13.9–15.6)	–0.06	0.06	0.95	0.85–1.06	0.34
Main presenting complaint	Gastroenteric (Intercept)	1.8 (1.2–2.5)	–3.99	0.05	0.02	0.02–0.02	
	Kidney disease	3.2 (2.4–4.1)	0.61	0.14	1.84	1.41–2.41	<0.01
	Other unwell	15.5 (15.0–16.0)	2.28	0.04	9.79	8.99–10.65	<0.01
	Pruritus	31.7 (30.7–32.8)	3.23	0.04	25.30	23.23–27.55	<0.01
	Respiratory	2.9 (2.3–3.6)	0.48	0.07	1.61	1.40–1.85	<0.01
	Trauma	6.6 (6.2–7.0)	1.32	0.05	3.75	3.43–4.11	<0.01
	Tumour	5.9 (5.5–6.4)	1.22	0.05	3.38	3.04–3.76	<0.01
Sex	Female (Intercept)	13.6 (13.3–14.0)	–1.87	0.02	0.15	0.15–0.16	
	Male	14.8 (14.4–15.2)	0.10	0.01	1.11	1.08–1.13	<0.01
Neuter status	Un-neutered (Intercept)	15.0 (14.6–15.4)	–1.76	0.02	0.17	0.17–0.18	
	Neutered	13.8 (13.4–14.2)	–0.10	0.01	0.91	0.88–0.93	<0.01
Microchip status	Un-microchipped (Intercept)	13.4 (13.1–13.8)	–1.89	0.02	0.15	0.15–0.16	
	Microchipped	14.9 (14.5–15.3)	0.13	0.01	1.14	1.11–1.16	<0.01
Vaccination status	Un-vaccinated (Intercept)	13.2 (12.9–13.6)	–1.90	0.02	0.15	0.14–0.16	
	Vaccinated	14.6 (14.3–15.0)	0.11	0.01	1.12	1.09–1.15	<0.01
Insurance status	Un-insured (Intercept)	14.5 (14.2–14.9)	–1.80	0.02	0.17	0.16–0.17	
	Insured	13.6 (13.2–14.1)	–0.07	0.01	0.93	0.91–0.96	<0.01
Owner urban status	Urban (Intercept)	14.4 (14.0–14.8)	–1.81	0.02	0.16	0.16–0.17	
	Rural	14.0 (13.6–14.4)	–0.04	0.02	0.97	0.94–1.00	0.02
Genetic breed group (*1*)	Retriever (Intercept)	15.3 (14.7–16.0)	–1.72	0.02	0.18	0.17–0.19	
	Crossbreed	13.3 (12.9–13.7)	–0.01	0.06	0.99	0.89–1.11	0.92
	Ancient / spitz	15.0 (13.5–16.5)	–0.16	0.02	0.85	0.82–0.89	<0.01
	Herding	8.2 (7.7–8.7)	–0.70	0.04	0.50	0.46–0.54	<0.01
	Mastiff-like	17.0 (16.4–17.6)	0.11	0.03	1.11	1.06–1.17	<0.01
	Scent hound	13.3 (12.2–14.3)	–0.18	0.04	0.83	0.77–0.91	<0.01
	Sight hound	5.3 (4.4–6.2)	–1.17	0.07	0.31	0.27–0.36	<0.01
	Small terrier	12.8 (12.3–13.3)	–0.22	0.02	0.80	0.76–0.84	<0.01
	Spaniel	16.1 (15.5–16.6)	0.04	0.02	1.04	0.99–1.08	0.13
	Toy	15.5 (14.7–16.3)	–0.02	0.03	0.99	0.92–1.05	0.64
	Unclassified	15.5 (14.9–16.1)	0.01	0.02	1.01	0.96–1.06	0.73
	Unknown	12.1 (10.8–13.4)	–0.29	0.06	0.75	0.66–0.85	<0.01
	Working dog	13.7 (12.9–14.5)	–0.13	0.03	0.88	0.82–0.93	<0.01
Practice type	Small animal (Intercept)	14.3 (13.9–14.8)	–1.81	0.02	0.16	0.16–0.17	
	Mixed	13.6 (12.9–14.3)	–0.08	0.04	0.92	0.85–1.00	0.05
	Small & equine	16.2 (14.3–18.2)	0.17	0.09	1.19	0.99–1.42	0.06
	Small & large	14.5 (13.6–15.4)	0.00	0.09	1.00	0.84–1.2	0.99
Accreditation	Not accredited (Intercept)	13.3 (12.3–14.4)	–1.90	0.05	0.15	0.14–0.16	
	1+ accredited site	14.4 (14.0–14.7)	0.09	0.05	1.10	1.00–1.20	0.05
Hospital status	No hospital site (Intercept)	14.0 (13.6–14.4)	–1.83	0.02	0.16	0.15–0.17	
	1+ hospital site	15.0 (14.3–15.7)	0.07	0.04	1.07	0.99–1.15	0.09
Referral interest	No (Intercept)	14.2 (13.9–14.6)	–1.83	0.02	0.16	0.16–0.17	
	Yes	14.2 (13.5–15.0)	0.03	0.03	1.03	0.96–1.1.0	0.47
Employed RCVS AVP†	None (Intercept)	13.9 (13.5–14.3)	–1.84	0.02	0.16	0.15–0.17	
	1+ AVP	15.3 (14.6–15.9)	0.08	0.04	1.08	1.01–1.16	0.03
Employed RCVS specialist†	None (Intercept)	14.3 (13.9–14.6)	–1.82	0.02	0.16	0.16–0.17	
	1+ specialist	12.0 (10.2–13.7)	–0.18	0.09	0.84	0.70–1.00+	0.05
		Continuous factors					
Age (years)	Intercept		–1.74	0.02	0.20	0.17–0.18	
	Age - linear		–0.32	0.01	0.73	0.71–0.75	<0.01
	Age - quadratic		–0.12	0.01	0.89	0.88–0.90	<0.01
	Age - cubic		0.03	0.01	1.03	1.02–1.04	<0.01
rIMD‡	Intercept		–1.82	0.02	0.16	0.16–0.17	<0.01
	rIMD		0.01	0.01	1.01	0.99–1.02	0.32
Dogs per household (*2*)	Intercept		–1.82	0.02	0.16	0.16–0.17	
	Dogs per household		–0.01	0.01	0.99	0.98–1.01	0.40
Dogs per km^2 (*2*)	Intercept		–1.82	0.02	0.16	0.16–0.17	
	Dogs per km		0.00	0.01	1.00	0.99–1.01	0.98

Variable	Category	% of prescribing consults (95% CI)	β	SE	OR	95% CI	*P*

*SE, standard error; OR, odds ratio.
†Royal College of Veterinary Surgeons (RCVS) Advanced Veterinary Practitioner (AVP) and / or specialist status.
‡Rescaled Indices of Multiple Deprivation (rIMD) quintile, 1 = most deprived.

Appendix Table 7. Descriptive summary of the percentage of total sick feline consultations where an animal was prescribed at least one antimicrobial (systemic, topical or systemic highest priority critically important (HPCIA) compared against animal breed, including breeds where in excess of 1,000 consultations were recorded*

Genetic breed group (*3*)	Cat breed	Systemic			Topical		Systemic HPCIA	
		n consults	%†	95% CI	%	95% CI	%	95% CI
Asian	Burmese	1,314	32.1	28.8–35.4	8.9	6.8–11.0	18.8	15.6–22.0
Asian	Siamese	1,814	35.3	31.9–38.7	5.0	3.9–6.2	17.6	14.8–20.4
Crossbreed	Crossbreed	93,599	32.9	31.9–33.8	5.7	5.5–5.9	17.2	16.1–18.4
Unclassified	Bengal	1,024	37.0	33.1–40.9	8.8	6.7–11.0	20.3	16.8–23.8
Unknown	Unknown	4,244	34.0	32.4–35.6	7.4	6.5–8.3	18.0	15.6–20.3
West Europe	British	2,707	29.1	26.1–32.2	9.5	7.3–11.6	14.6	12.2–17.0
West Europe	Persian	1,870	29.9	26.6–33.2	11.0	9.3–12.7	16.1	13.4–18.8

*HPCIA, highest priority critically important antimicrobial.
†Percentage of consultations where at least one antimicrobial was prescribed.

Appendix Table 8. Descriptive summary of the percentage of total sick feline consultations prescribed a systemic antimicrobial. Also included are parameter estimates from a series of univariable mixed effect logistic regression models assessing the association between a range of animal, owner, practitioner and practice-related factors and the probability of prescribing a systemic antimicrobial. Random effects include animal, site, and practice.

Variable	Category	% of prescribing consults (95% CI [a])	β	SE	OR	95% CI	*P*
Categorical factors							
Country	England (Intercept)	32.5 (31.5–33.5)	–0.77	0.03	0.46	0.44–0.49	
	Scotland	37.0 (33.9–40.1)	0.06	0.09	1.06	0.90–1.26	0.47
	Wales	33.4 (29.9–37.0)	0.34	0.10	1.40	1.15–1.71	<0.01
Main presenting complaint	Gastroenteric (Intercept)	30.5 (28.1–32.9)	–0.83	0.04	0.44	0.41–0.47	
	Kidney disease	20.7 (18.8–22.6)	–0.47	0.05	0.62	0.56–0.69	<0.01
	Other unwell	27.2 (26.1–28.2)	–0.20	0.03	0.82	0.78–0.87	<0.01
	Pruritus	26.8 (24.9–28.7)	–0.23	0.03	0.79	0.74–0.85	<0.01
	Respiratory	53.0 (50.6–55.4)	0.91	0.04	2.49	2.32–2.69	<0.01
	Trauma	53.5 (52.3–54.7)	0.99	0.03	2.68	2.53–2.84	<0.01
	Tumour	20.7 (19.0–22.3)	–0.58	0.05	0.56	0.51–0.62	<0.01
Sex	Female (Intercept)	30.1 (29.1–31.1)	–0.88	0.03	0.42	0.39–0.44	
	Male	35.4 (34.4–36.4)	0.26	0.02	1.30	1.26–1.34	<0.01
Neuter status	Un-neutered (Intercept)	33.1 (31.9–34.2)	–0.74	0.03	0.48	0.45–0.51	
	Neutered	32.8 (31.8–33.8)	–0.00	0.02	1.00	0.96–1.04	0.87
Microchip status	Un-microchipped (Intercept)	32.2 (31.3–33.2)	–0.80	0.03	0.45	0.43–0.48	
	Microchipped	33.9 (32.8–35.1)	0.14	0.02	1.15	1.12–1.19	<0.01
Vaccination status	Un-vaccinated (Intercept)	33.6 (32.6–34.6)	–0.73	0.03	0.48	0.46–0.51	
	Vaccinated	32.2 (31.2–33.2)	–0.03	0.02	0.97	0.94–1.00	0.05
Insurance status	Un-insured (Intercept)	33.8 (32.8–34.8)	–0.71	0.03	0.49	0.47–0.52	
	Insured	28.9 (27.7–30.1)	–0.20	0.02	0.82	0.79–0.86	<0.01
Owner urban status	Urban (Intercept)	32.1 (31.1–33.1)	–0.76	0.03	0.47	0.44–0.50	
	Rural	34.8 (33.3–36.2)	0.04	0.02	1.05	1.00–1.09	0.04
Genetic breed group (*3*)	West Europe (Intercept)	30.8 (29.1–32.4)	–0.88	0.04	0.41	0.38–0.45	
	Asian	33.1 (30.7–35.5)	0.14	0.05	1.15	1.04–1.27	0.01
	Crossbreed	32.9 (31.9–33.8)	0.14	0.03	1.16	1.0–1.23	<0.01
	Mediterranean	42.5 (27.5–57.4)	0.48	0.26	1.61	0.97–2.67	0.06
	Unclassified	34.7 (32.2–37.3)	0.22	0.06	1.25	1.11–1.39	<0.01
	Unknown	34.0 (32.4–35.6)	0.18	0.05	1.19	1.08–1.31	<0.01
Practice type	Small animal (Intercept)	32.2 (31.1–33.2)	–0.79	0.03	0.45	0.43–0.48	
	Mixed	35.4 (32.8–38.0)	0.24	0.08	1.27	1.10–1.47	<0.01
	Small & equine	28.7 (22.4–35.0)	–0.02	0.17	0.98	0.70–1.38	0.90
	Small & large	37.7 (32.7–42.7)	0.25	0.16	1.29	0.94–1.76	0.11
Accreditation	Not accredited (Intercept)	36.4 (34.2–38.6)	–0.54	0.08	0.58	0.50–0.68	
	1+ accredited site	32.2 (31.2–33.2)	–0.23	0.09	0.80	0.67–0.94	<0.01
Hospital status	No hospital site (Intercept)	33.3 (32.2–34.4)	–0.72	0.03	0.49	0.46–0.52	
	1+ hospital site	31.2 (29.5–32.9)	–0.13	0.07	0.88	0.76–1.01	0.07
Referral interest	No (Intercept)	33.2 (32.1–34.3)	–0.71	0.03	0.49	0.46–0.53	
	Yes	31.9 (30.1–33.8)	–0.11	0.06	0.90	0.79–1.01	0.08
Employed RCVS AVP†	None (Intercept)	33.4 (32.3–34.5)	–0.71	0.03	0.49	0.46–0.53	

	1+ AVP	31.3 (29.4–33.2)	–0.18	0.07	0.84	0.73–0.96	0.01
Employed RCVS specialist†	None (Intercept)	32.9 (32.0–33.9)	–0.74	0.03	0.48	0.45–0.51	
	1+ specialist	29.0 (24.7–33.4)	–0.14	0.17	0.87	0.62–1.21	0.41
		Continuous factors					
Age (years)	Intercept		–0.64	0.03	0.53	0.50–0.56	
	Age - linear		–0.53	0.02	0.59	0.57–0.61	<0.01
	Age - quadratic		–0.13	0.01	0.87	0.86–0.89	<0.01
	Age - cubic		0.12	0.01	1.13	1.11–1.15	<0.01
rIMD [f]	Intercept		–0.74	0.03	0.48	0.45–0.50	
	IMD		–0.03	0.01	0.97	0.96–0.99	<0.01
Cats per household	Intercept		–0.74	0.03	0.48	0.45–0.50	
	Cats per household		–0.00	0.01	1.00	0.97–1.02	0.73
Cats per km^2 (*2*)	Intercept		–0.74	0.03	0.48	0.45–0.50	
	Cats per km		–0.02	0.01	0.98	0.97–1.00	0.02

*SE, standard error; OR, odds ratio.
†Royal College of Veterinary Surgeons (RCVS) Advanced Veterinary Practitioner (AVP) and / or specialist status.
‡Rescaled Indices of Multiple Deprivation (rIMD) quintile, 1 = most deprived.

Appendix Table 9. Descriptive summary of the percentage of total sick feline consultations prescribed a systemic highest priority critically important antimicrobial (HPCIA). Also included are parameter estimates from a series of univariable mixed effect logistic regression models assessing the association between a range of animal, owner, practitioner and practice-related factors and the probability of prescribing a systemic HPCIA. Random effects include animal, site, and practice.

Variable	Category	% of prescribing consults (95% CI)	β	SE	OR	95% CI	*P*
Categorical factors							
Country	England (Intercept)	17.1 (16.0–18.1)	–1.71	0.06	0.18	0.16–0.20	
	Scotland	17.5 (9.8–25.3)	0.07	0.12	1.07	0.86–1.35	0.54
	Wales	18.0 (14.9–21.1)	0.18	0.17	1.20	0.86–1.68	0.29
Main presenting complaint	Gastroenteric (Intercept)	6.9 (5.9–7.9)	–2.71	0.07	0.07	0.06–0.08	
	Kidney disease	13.7 (11.9–15.5)	0.75	0.07	2.12	1.84–2.44	<0.01
	Other unwell	14.2 (13.2–15.2)	0.79	0.05	2.20	2.02–2.41	<0.01
	Pruritus	19.8 (18.1–21.5)	1.17	0.05	3.23	2.92–3.57	<0.01
	Respiratory	29.4 (26.9–31.9)	1.72	0.05	5.57	5.00–6.19	<0.01
	Trauma	27.1 (24.6–29.5)	1.68	0.05	5.35	4.88–5.87	<0.01
	Tumour	12.3 (11.0–13.7)	0.57	0.07	1.77	1.55–2.01	<0.01
Sex	Female (Intercept)	16.3 (15.2–17.4)	–1.76	0.06	0.17	0.15–0.19	
	Male	17.9 (16.7–19.1)	0.13	0.02	1.14	1.10–1.18	<0.01
Neuter status	Un-neutered (Intercept)	16.3 (15.0–17.7)	–1.78	0.06	0.17	0.15–0.19	
	Neutered	17.3 (16.2–18.4)	0.10	0.03	1.11	1.06–1.16	<0.01
Microchip status	Un-microchipped (Intercept)	16.8 (15.7–17.9)	–1.73	0.06	0.18	0.16–0.20	
	Microchipped	17.6 (16.4–18.8)	0.08	0.02	1.09	1.05–1.13	<0.01
Vaccination status	Un-vaccinated (Intercept)	17.5 (16.3–18.7)	–1.67	0.06	0.19	0.17–0.21	
	Vaccinated	16.8 (15.7–17.8)	–0.05	0.02	0.95	0.91–0.98	<0.01
Insurance status	Un-insured (Intercept)	17.6 (16.5–18.8)	–1.67	0.06	0.19	0.17–0.21	
	Insured	15.0 (13.7–16.2)	–0.13	0.03	0.88	0.84–0.93	<0.01
Owner urban status	Urban (Intercept)	16.5 (15.4–17.6)	–1.71	0.06	0.18	0.16–0.20	
	Rural	18.7 (16.9–20.5)	0.06	0.03	1.06	1.01–1.11	0.03
Genetic breed group (*3*)	West Europe (Intercept)	15.3 (13.8–16.9)	–1.88	0.07	0.15	0.13–0.17	
	Asian	17.2 (15.2–19.3)	0.19	0.07	1.21	1.06–1.37	<0.01
	Crossbreed	17.2 (16.1–18.3)	0.20	0.04	1.23	1.13–1.33	<0.01
	Mediterranean	22.0 (7.1–36.9)	0.11	0.32	1.12	0.60–2.09	0.73
	Unclassified	16.6 (14.7–18.6)	0.15	0.07	1.16	1.01–1.34	0.04
	Unknown	18.0 (15.6–20.3)	0.14	0.06	1.15	1.02–1.30	0.02
Practice type	Small animal (Intercept)	16.5 (15.3–17.8)	–1.73	0.06	0.18	0.16–0.20	
	Mixed	18.8 (16.1–21.5)	0.10	0.16	1.11	0.81–1.50	0.52
	Small & equine	18.2 (12.7–23.7)	0.27	0.37	1.30	0.64–2.67	0.47
	Small & large	20.1 (14.4–25.9)	0.28	0.32	1.32	0.71–2.46	0.38
Accreditation	Not accredited (Intercept)	14.5 (10.5–18.4)	–1.93	0.16	0.15	0.11–0.20	
	1+ accredited site	17.7 (16.6–18.7)	0.27	0.17	1.31	0.93–1.83	0.12
Hospital status	No hospital site (Intercept)	17.0 (15.7–18.4)	–1.67	0.06	0.19	0.17–0.21	
	1+ hospital site	17.4 (15.6–19.1)	–0.14	0.15	0.87	0.65–1.16	0.34
Referral interest	No (Intercept)	17.5 (16.1–18.8)	–1.67	0.07	0.19	0.17–0.22	
	Yes	16.2 (14.3–18.2)	–0.08	0.12	0.92	0.72–1.17	0.50
Employed RCVS AVP†	None (Intercept)	17.3 (15.9–18.6)	–1.69	0.06	0.19	0.16–0.21	
	1+ AVP	16.8 (14.8–18.7)	–0.04	0.14	0.96	0.73–1.27	0.77

Variable	Category	% of prescribing consults (95% CI)	β	SE	OR	95% CI	*P*
Employed RCVS specialist†	None (Intercept)	17.1 (16.0–18.2)	–1.70	0.06	0.18	0.16–0.21	
	1+ specialist	16.5 (12.5–20.5)	0.06	0.34	1.07	0.55–2.06	0.85
Continuous factors							
Age (years)	Intercept		–1.51	0.06	0.22	0.20–0.25	
	Age - linear		–0.38	0.02	0.68	0.66–0.71	<0.01
	Age - quadratic		–0.20	0.01	0.82	0.80–0.83	<0.01
	Age - cubic		0.17	0.01	1.18	1.16–1.20	<0.01
rIMD‡	Intercept		–1.69	0.06	0.18	0.17–0.21	
	IMD		0.00	0.01	1.00	0.98–1.03	0.83
Cats per household (*2*)	Intercept		–1.69	0.06	0.18	0.17–0.21	
	Cats per household		0.01	0.02	1.01	0.98–1.04	0.54
Cats per km^2 (*2*)	Intercept		–1.69	0.06	0.18	0.16–0.21	
	Cats per km		–0.01	0.01	0.99	1.00–1.01	0.28

*SE, standard error; OR, odds ratio.
†Royal College of Veterinary Surgeons (RCVS) Advanced Veterinary Practitioner (AVP) and / or specialist status.
‡Rescaled Indices of Multiple Deprivation (rIMD) quintile, 1 = most deprived.

Appendix Table 10. Descriptive summary of the percentage of total sick feline consultations prescribed a topical antimicrobial. Also included are parameter estimates from a series of univariable mixed effect logistic regression models assessing the association between a range of animal, owner, practitioner and practice-related factors and the probability of prescribing a topical antimicrobial. Random effects include animal, site, and practice.

Variable	Category	% of prescribing consults (95% CI)	β	SE	OR	95% CI	*P*
Categorical factors							
Country	England (Intercept)	6.0 (5.8–6.3)	–2.77	0.02	0.06	0.06–0.07	
	Scotland	6.6 (5.5–7.7)	0.07	0.09	1.07	0.90–1.28	0.45
	Wales	6.6 (6.0–7.2)	0.14	0.09	1.15	0.97–1.36	0.11
Main presenting complaint	Gastroenteric (Intercept)	1.1 (0.6–1.6)	–4.47	0.10	0.01	0.01–0.01	
	Kidney disease	0.8 (0.5–1.1)	–0.34	0.23	0.72	0.46–1.11	0.14
	Other unwell	7.1 (6.8–7.4)	1.89	0.10	6.59	5.40–8.04	<0.01
	Pruritus	10.8 (10.0–11.7)	2.35	0.11	10.49	8.54–12.89	<0.01
	Respiratory	5.7 (4.9–6.4)	1.63	0.12	5.10	4.07–6.40	<0.01
	Trauma	4.6 (4.3–5.0)	1.43	0.11	4.20	3.41–5.17	<0.01
	Tumour	1.7 (1.3–2.1)	0.42	0.16	1.53	1.13–2.08	0.01
Sex	Female (Intercept)	6.0 (5.7–6.2)	–2.78	0.03	0.06	0.06–0.07	
	Male	6.2 (6.0–6.5)	0.04	0.03	1.05	0.99–1.10	0.11
Neuter status	Un-neutered (Intercept)	7.3 (6.8–7.7)	–2.57	0.04	0.08	0.07–0.08	
	Neutered	5.9 (5.6–6.1)	–0.23	0.03	0.79	0.74–0.85	<0.01
Microchip status	Un-microchipped (Intercept)	5.9 (5.7–6.2)	–2.79	0.03	0.06	0.06–0.07	
	Microchipped	6.4 (6.0–6.7)	0.09	0.03	1.09	1.03–1.16	<0.01
Vaccination status	Un-vaccinated (Intercept)	6.2 (5.9–6.5)	–2.74	0.03	0.06	0.06–0.07	
	Vaccinated	6.0 (5.8–6.3)	–0.02	0.03	0.98	0.93–1.03	0.42
Insurance status	Un-insured (Intercept)	6.3 (6.1–6.5)	–2.72	0.02	0.07	0.06–0.07	
	Insured	5.3 (4.9–5.8)	–0.19	0.04	0.83	0.77–0.89	<0.01
Owner urban status	Urban (Intercept)	6.0 (5.8–6.3)	–2.77	0.03	0.06	0.06–0.07	
	Rural	6.3 (5.9–6.7)	0.04	0.03	1.04	0.97–1.11	0.26
Genetic breed group (*3*)	West Europe (Intercept)	9.5 (8.4–10.6)	–2.28	0.05	0.10	0.09–0.11	
	Asian	6.9 (5.8–8.0)	–0.29	0.09	0.75	0.63–0.88	<0.01
	Crossbreed	5.7 (5.5–5.9)	–0.54	0.05	0.58	0.53–0.64	<0.01
	Mediterranean	5.5 (0.5–10.4)	–0.47	0.49	0.62	0.24–1.64	0.34
	Unclassified	8.3 (7.2–9.5)	–0.13	0.09	0.88	0.74–1.05	0.17
	Unknown	7.4 (6.4–8.3)	–0.29	0.08	0.75	0.64–0.88	<0.01
Practice type	Small animal (Intercept)	6.0 (5.8–6.3)	–2.77	0.03	0.06	0.06–0.07	
	Mixed	6.4 (5.9–7.0)	0.07	0.06	1.08	0.96–1.21	0.20
	Small & equine	5.7 (4.5–6.8)	–0.11	0.14	0.89	0.68–1.18	0.43
	Small & large	6.4 (5.5–7.3)	0.09	0.12	1.09	0.86–1.39	0.47
Accreditation	Not accredited (Intercept)	5.9 (5.2–6.5)	–2.77	0.06	0.06	0.06–0.07	
	1+ accredited site	6.2 (5.9–6.4)	0.02	0.07	1.02	0.90–1.16	0.74
Hospital status	No hospital site (Intercept)	6.0 (5.7–6.2)	–2.76	0.03	0.06	0.06–0.07	
	1+ hospital site	6.5 (6.1–6.9)	0.04	0.06	1.05	0.94–1.16	0.42
Referral interest	No (Intercept)	6.0 (5.8–6.3)	–2.78	0.03	0.06	0.06–0.07	
	Yes	6.3 (5.9–6.8)	0.08	0.05	1.08	0.98–1.19	0.10
Employed RCVS AVP†	None (Intercept)	6.1 (5.9–6.4)	–2.75	0.03	0.06	0.06–0.07	
	1+ AVP	6.0 (5.6–6.4)	–0.03	0.05	0.97	0.87–1.08	0.57

Employed RCVS specialist†	None (Intercept)	6.1 (5.9–6.3)	–2.75	0.02	0.06	0.06–0.07	
	1+ specialist	5.3 (4.1–6.6)	–0.13	0.14	0.88	0.66–1.16	0.36
Continuous factors							
Age (years)	Intercept		–2.86	0.03	0.06	0.05–0.06	
	Age - linear		–0.29	0.03	0.75	0.70–0.79	<0.01
	Age - quadratic		0.04	0.02	1.04	1.01–1.08	0.01
	Age - cubic		–0.04	0.02	0.96	0.93–0.99	0.01
rIMD‡	Intercept		–2.76	0.02	0.06	0.06–0.07	
	IMD		–0.04	0.02	0.96	0.93–0.99	0.01
Cats per household (*2*)	Intercept		–2.75	0.02	0.06	0.06–0.07	
	Cats per household		0.01	0.02	1.01	0.97–1.04	0.72
Cats per km^2 (*2*)	Intercept		–2.75	0.02	0.06	0.06–0.07	
	Cats per km		0.01	0.01	1.01	0.98–1.03	0.72

*SE, standard error; OR, odds ratio.
†Royal College of Veterinary Surgeons (RCVS) Advanced Veterinary Practitioner (AVP) and / or specialist status.
‡Rescaled Indices of Multiple Deprivation (rIMD) quintile, 1 = most deprived.

References

1. vonholdt BM, Pollinger JP, Lohmueller KE, Han E, Parker HG, Quignon P, et al. Genome-wide SNP and haplotype analyses reveal a rich history underlying dog domestication. Nature. 2010;464:898–902. PubMed https://doi.org/10.1038/nature08837

2. Aegerter J, Fouracre D, Smith GC. A first estimate of the structure and density of the populations of pet cats and dogs across Great Britain. PLoS One. 2017;12:e0174709. PubMed https://doi.org/10.1371/journal.pone.0174709

3. 31. Lipinski MJ, Froenicke L, Baysac KC, Billings NC, Leutenegger C, Levy AM, et al. The ascent of cat breeds: genetic evaluations of breeds and worldwide random-bred populations. Genomics. 2008;91:12–1. https://doi.org/10.1016/j.ygeno.2007.10.009
